# Single Nucleotide Polymorphisms Associated with Colorectal Cancer Susceptibility and Loss of Heterozygosity in a Taiwanese Population

**DOI:** 10.1371/journal.pone.0100060

**Published:** 2014-06-26

**Authors:** Chih-Yung Yang, Ruey-Hwa Lu, Chien-Hsing Lin, Chih-Hung Jen, Chien-Yi Tung, Shung-Haur Yang, Jen-Kou Lin, Jeng-Kai Jiang, Chi-Hung Lin

**Affiliations:** 1 Institute of Microbiology and Immunology, National Yang-Ming University, Taipei, Taiwan; 2 Department of Education and Research, Taipei City Hospital, Taipei, Taiwan; 3 Department of Surgery, Taipei City Hospital, Taipei, Taiwan; 4 Division of Molecular and Genomic Medicine, National Health Research Institutes, Zhunan, Taiwan; 5 VYM Genome Research Center, National Yang-Ming University, Taipei, Taiwan; 6 Department of Surgery, Taipei Veterans General Hospital and School of Medicine, National Yang-Ming University, Taipei, Taiwan; Ohio State University Medical Center, United States of America

## Abstract

Given the significant racial and ethnic diversity in genetic variation, we are intrigued to find out whether the single nucleotide polymorphisms (SNPs) identified in genome-wide association studies of colorectal cancer (CRC) susceptibility in East Asian populations are also relevant to the population of Taiwan. Moreover, loss of heterozygosity (LOH) may provide insight into how variants alter CRC risk and how regulatory elements control gene expression. To investigate the racial and ethnic diversity of CRC-susceptibility genetic variants and their relevance to the Taiwanese population, we genotyped 705 CRC cases and 1,802 healthy controls (Taiwan Biobank) for fifteen previously reported East Asian CRC-susceptibility SNPs and four novel genetic variants identified by whole-exome sequencing. We found that rs10795668 in *FLJ3802842* and rs4631962 in *CCND2* were significantly associated with CRC risk in the Taiwanese population. The previously unreported rs1338565 was associated with a significant increased risk of CRC. In addition, we also genotyped tumor tissue and paired adjacent normal tissues of these 705 CRC cases to search for LOH, as well as risk-associated and protective alleles. LOH analysis revealed preferential retention of three SNPs, rs12657484, rs3802842, and rs4444235, in tumor tissues. rs4444235 has been recently reported to be a cis-acting regulator of *BMP4* gene; in this study, the C allele was preferentially retained in tumor tissues (p = 0.0023). rs4631962 and rs10795668 contribute to CRC risk in the Taiwanese and East Asian populations, and the newly identified rs1338565 was specifically associated with CRC, supporting the ethnic diversity of CRC-susceptibility SNPs. LOH analysis suggested that the three CRC risk variants, rs12657484, rs3802842, and rs4444235, exhibited somatic allele-specific imbalance and might be critical during neoplastic progression.

## Introduction

Colorectal cancer (CRC) affects 1.23 million people worldwide and causes 0.6 million deaths annually; it is becoming the most frequently diagnosed cancer in developed countries [1]. During the last two decades, the incidence of CRC has increased dramatically in developed Asian countries, including Japan, Hong Kong, Singapore, Korea, and Taiwan, and is now comparable to that of Western countries [2], [3]. In Taiwan, CRC has been the most frequently diagnosed cancer since 2007 [4], [5]. A number of genetic and environmental factors are known to cause CRC [6]–[9].

There is a direct association between sporadic tumor occurrence and susceptibility variants carried by an individual. Two percent of the European population carries multiple inherited low-risk alleles that increase the rate of CRC incidence about four-fold [10], [11]. Over the past two decades, many candidate gene studies have evaluated common genetic risk factors for CRC; however, only a few of these have been replicated in subsequent studies [12]. Recent genome-wide association studies (GWAS) identified 15 common genetic susceptibility loci for CRC [13]–[21]; however, less than 15% of CRC heritability could be explained by these newly identified genetic factors including known high-penetrance variations in CRC susceptibility genes [13], [14].

Neoplastic progression is often associated with accumulation of somatic-cell genetic changes as the tumor progresses [13]–[20]. Loss of heterozygosity (LOH) can be caused by mutation of one allele and loss of another allele through mitotic nondisjunction, chromosome nondisjunction, or physical deletion, followed by reduplication of the remaining mitotic, chromosome recombination, and gene conversion [21]–[23]. Identification of genome-wide LOH patterns in tumors may reveal the specific region that anchors tumor suppressor genes and suggest novel molecular mechanisms for carcinogenesis.

GWAS identify SNPs that tag linkage disequilibrium (LD) blocks in the genome, thus capturing a large proportion of common genetic variations. Fifteen SNPs associated with CRC in the East Asian populations (rs6687758, rs10936599, rs647161, rs10505477, rs6983267, rs7014346, rs10795668, rs1665650, rs3802842, rs107742124, rs4444235, rs4779584, rs9929218, rs4939827 and rs961253) were evaluated in a Taiwanese population [24]. The control minor allele frequency of rs10774214, rs647161, and rs16656650 in 653,291 SNPs on Taiwan-specific customized SNP array (Affymetrix Inc.) were not available in the Taiwan BioBank database; we searched in Taiwan Biobank and identified rs4631962, rs12657484, and rs1665645, which are in strong LD (r^2^≥0.8) with the original SNPs.

In this study, the tumor and adjacent non-tumor tissues of 705 colorectal cancer patients in Taiwan were collected and genotyped in an attempt to replicate GWAS findings and evaluate the possibility of the allele-specific imbalance in tumor tissues. We found a novel SNP (rs1338565) and two published SNPs (rs10795668 and rs464631962) associated with CRC risk and three SNPs (rs12657484, rs3802842, and rs4444235) showing statistical significance in allele specific retention in tumors.

## Materials and Methods

### Study population and DNA preparation

The study included a population-based series of 705 paired CRC tumors and adjacent normal tissues collected since 2006 at Taipei Veterans General Hospitals. All tissue samples of FFPE were diagnosed by experienced pathologists. The tumor tissues consist of 50% or more tumor cells for collecting DNA. Germline DNA extracted from RNAlater-immersed adjacent normal colonic tissue and corresponding tumor DNA were available. The 1802 control subjects were anonymous healthy blood donors from the Taiwan Biobank (http://taiwanview.twbiobank.org.tw/taiwanview/search.do). Written informed consent was obtained from all patients and the study was approved by Institutional Review Board of Taipei Veterans General Hospital, Taipei, Taiwan. The genomic DNA of paired tumor and adjacent normal tissues were extracted using QIAamp Mini Kit according to the manufacturer’s protocols (Qiagen).

### Exome Library Preparation and Sequencing

Exome sequence capture was performed following the procedure provided for the Agilent SureSelect Platform (SureSelect Human All Exon V4 kit). The captured library was performed with paired-end 90 base reads on the Illumina HiSeq 2000 platform. The average sequencing depth is more than 100-fold, and the coverage of target region is at least 99%.

### Sequencing Data analysis: Alignment, Variant Calling, and Annotation

The adapter sequence in the raw data was removed, and low quality reads were discarded. Sequence reads were aligned to the reference genome (hg19) using the BWA program [25]. The alignment information were stored in BAM format files and for further processing, along with fixing mate-pair information to add read group information and marking duplicate reads caused by polymerase chain reaction. The variant calling and annotation of the processed BAM files were performed using different programs. Single Nucleotide Polymorphisms (SNPs) were detected by SOAPsnp [26] and small Insertion/Deletions (InDels) are detected by SAMtools and Single Nucleotide Variants (SNVs) were detected by Varscan [27] and somatic InDels were detected by GATK [28].

### SNP selection and genotyping

Nineteen CRC- susceptibility SNPs were genotyped in all samples, and among them, 15 were reported to be directly or indirectly (LD γ2>0.8) associated with CRC susceptibility in East Asians [24]. For clarifying population stratification within cases and controls, 44 frequently-used unlinked SNPs were also genotyped in all cases and controls [29]. SNP genotyping were carried out by using the Sequenom MassARRAY system. The PCR and single-base extension primers were designed by using the MassARRAY Assay Design 3.1 software (Sequenom, San Diego, CA). PCR reactions in a final volume of 5 µl contained 1 pmol of the corresponding primers, 5 ng genomic DNA, and reaction mix (Sequenom) in 384-well plates. PCR conditions were as follows: 94°C for 15 min, followed by 40 cycles of 94°C (20 s), 56°C (30 s), 72°C (60 s), and a final extension of 72°C for 3 min. In the primer extension procedure, each sample was denatured at 94°C, followed by 40 cycles of 94°C (5 s), 52°C (5 s), 72°C (5 s). The mass spectrum from time-resolved spectra was retrieved by using a MassARRAY mass spectrometer (Sequenom), and each spectrum was then analyzed using the Sequenom Typer 4.0 software (Sequenom) to perform the SNP genotype calling.

### Statistical analyses

Pearson’s χ2 tests were used to compare the difference of SNP allele and genotype frequencies between cases and well-match controls. Hardy–Weinberg equilibrium of each SNP was tested by goodness-of-fit χ2 test to compare the expected frequency of genotypes in controls. The effects of polymorphisms on the risk of colorectal cancer were expressed as odds ratios (ORs) with 95% confidence intervals (95% CIs), evaluated using unconditional logistic regression analysis. To identify allele-specific imbalance, the genotype of each SNP (called based on the Sequenom default algorithm) was compared in tumor and adjacent non-tumor tissues, and only patients with germline SNP heterozygous calls were used for following analysis. Fisher’s exact test was used for analyzing SNP LOH in cancer tissues. Principal component analysis (PCA) was used to clarify population stratification using 44 unlinked SNPs. Bonferroni and permutation methods were used to adjust p-values in multiple comparisons. All the above statistical analyses were performed using SAS/STAT version 8 software (SAS Institute, Cary, NC, USA).

## Results

### CRC-susceptibility SNP association analysis

Two resources of CRC candidates SNPs were used in this study. One was selected from previous East Asian CRC-susceptibility association article [24], and another is identified based on the exomic-sequencing data in few tumor tissues in this study. Based on the results of previous study [24], 15 SNPs, including rs6687758, rs10936599, rs647161, rs10505477, rs6983267, rs7014346, rs10795668, rs1665650, rs3802842, rs107742124, rs4444235, rs4779584, rs9929218, rs4939827 and rs961253, were associated with CRC susceptibility in East Asian population. Although only three SNPs showed more statistical associations with CRC susceptibility after Bonferroni strict p-value adjusted (rs6983267, rs10795668, rs4939827). Due to the current limitation of public Taiwan Biobank, three of these SNPs (3/15) were not included in the database. We selected rs4631962, rs12657484, and rs1665645 to represent rs10774214, rs647161, and rs16656650, respectively, due to the strong LD (r^2^≥0.8) between them.

We originally searched a set of genes and mutations in colorectal polyps to assess patients’ subsequent carcinogenic risk. We performed exomic-sequencing experiments on paired tissues from seven CRC patients (i.e. cancer tissues, cancer-synchronous polyps, and adjacent normal tissues) and six cases of incidental, non-cancer-related polyps. After differential analysis, we selected 62 CRC carcinogenesis-related gene variations present in tumors and cancer-synchronous polyp but absent in normal tissues and incidental polyps to be verified in 47 pairs of CRC tumor and paired normal adjacent tissues by Sequenom MassArray (data not shown). We identified four candidate SNPs associated with CRC susceptibility in a comparison with 1802 healthy controls from the Taiwan Biobank.

**Figure 1:**
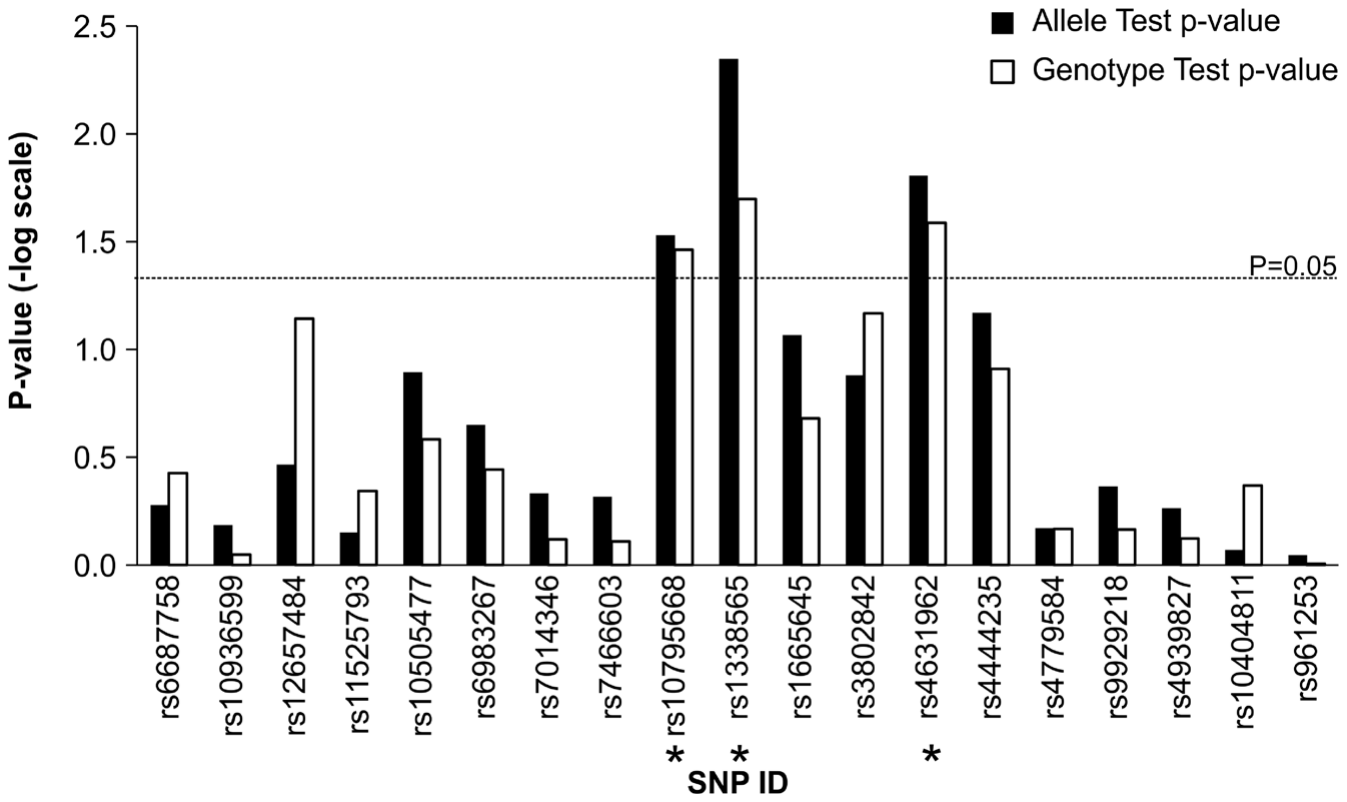
Statistical significances of colorectal cancer-susceptibility SNPs in Taiwan population. Fifteen reported and four newly-identified CRC-susceptibility SNPs were genotyped in 705 cases and compared to 1802 healthy control in Taiwan View Biobank. Allele and genotype frequencies were compared using χ2 tests. Red dash line indicates the p = 0.05. The asterisk indicates significant p-value.

We combined 15 reported and 4 newly-identified SNPs to genotype these SNPs in 705 independent CRC pairs of tumor and paired normal adjacent tissues. Although Taiwan Biobank is a database which randomly enrolled samples in the population and serves as good and public controls for other projects. Due to population stratification in these CRC patients and controls, we genotyped 44 frequently used unlinked SNPs (Table S1 in File S1) and performed PCA analysis. There was no population stratification within these samples (Figure S1 in File S1), and the estimated inflation factor (λ = 1.001) was very small, indicating cases are genetically matched with the public control database. Nineteen SNPs were genotyped by using the MassARRAY system in the non-tumor tissue of 705 CRC patients, and compared to the data of 1,802 controls. As shown in Figure 1, we found a significant CRC risk association (unadjusted p-value <0.05) for rs10795668 (OR = 1.13, unadjusted p-value = 0.03) in FLJ3802842, rs4631962 (OR = 1.143, unadjusted p-value = 0.016) in CCND2 and the newly-identified rs1338565 (OR = 1.16, unadjusted p-value = 0.005) in allele- and genotype-based analyses. For the purpose of replicating these known CRC susceptibility SNPs, Bonferroni p-value adjustment was perhaps too strict because the sample size of CRC patients in this study was marginal and these SNPs were correlated with CRC. We used permutation method (bootstrap n = 1,000) to adjust the p-values, and rs4631962 (adjusted p-value = 0.043) and rs1338565 (adjusted p-value = 0.015) showed marginal significance of allele frequency differences between 705 cases and 1,802 controls (Table 1). One of purpose of this study was to evaluate and identify SNPs which are strongly susceptible to CRC in Taiwan’s population, so the sample size of this study is large enough (>0.8) to identify SNP with large effect size (allele frequency difference>0.05, type I error = 0.05) in case-control association study. However, only 705 cases and 1,802 controls might be insufficient to identify CRC-susceptibility SNPs with small effect sizes.

**Table 1:** Replication of colorectal cancer-susceptibility SNPs in Taiwan population.

rs#	Chr	Position	Case MAF	Control MAF	Allele Test p-value (adjusted†)	Odds Ratio (OR)	95% OR	HW Test p-value	Published study unadjusted p-value‡
rs6687758	1	222164948	0.203	0.196	0.529 (0.878)	1.042	0.895–1.212	0.77	0.005
rs10936599	3	169492101	0.451	0.457	0.654 (0.795)	1.024	0.906–1.156	0.67	0.03
rs12657484	5	134503751	0.331	0.342	0.344 (0.654)	1.055	0.926–1.200	0.27	NA
rs11525793	7	15648935	0.407	0.412	0.71 (0.891)	1.02 0	0.901–1.154	0.59	NA
rs10505477	8	128407443	0.446	0.426	0.128 (0.315)	1.083	0.959–1.224	0.63	0.005
rs6983267	8	128413305	0.444	0.428	0.225 (0.415)	1.066	0.943–1.204	0.5	8.5×10^–5^
rs7014346	8	128424792	0.324	0.333	0.466 (0.571)	1.042	0.915–1.185	0.83	0.016
rs7466603	9	94483198	0.072	0.077	0.484 (0.647)	1.073	0.850–1.354	0.13	NA
*rs10795668	10	8701219	0.35	0.378	0.03 (0.087)	1.126	0.992–1.278	1	3.84×10^–9^
*rs1338565	10	44059676	0.439	0.476	0.005 (0.015)	1.160	1.027–1.311	0.17	NA
rs1665645	10	118487954	0.288	0.309	0.086 (0.124)	1.104	0.966–1.261	0.25	NA
rs3802842	11	111171709	0.473	0.453	0.133 (0.216)	1.081	0.958–1.221	0.6	0.021
*rs4631962	12	4373132	0.328	0.299	0.016 (0.043)	1.143	1.004–1.302	0.34	NA
rs4444235	14	54410919	0.473	0.497	0.068 (0.101)	1.100	0.974–1.242	0.28	0.025
rs4779584	15	32994756	0.192	0.197	0.677 (0.715)	1.028	0.882–1.198	0.46	0.002
rs9929218	16	68820946	0.187	0.195	0.434 (0.491)	1.054	0.903–1.230	0.71	0.01
rs4939827	18	46453463	0.304	0.297	0.548 (0.637)	1.034	0.907–1.180	0.37	2.86×10^–4^
rs10404811	19	58154807	0.368	0.366	0.856 (0.861)	1.010	0.873–1.123	0.48	NA
rs961253	20	6404281	0.076	0.075	0.903 (0.921)	1.012	0.786–1.243	1	0.03

Note: Fifteen reported and four newly-identified CRC-susceptibility SNPs were genotyped in 705 cases and compared to 1802 healthy control in Taiwan View Biobank. Allele and genotype frequencies were compared using Chi-square tests.

*SNP showed significant differences in allele frequencies between cases and controls (unadjusted p-value<0.05).

† Permutation-based p-value adjustments (bootstrap n = 1,000) was applied.

‡ Jia WH *et al. Nature Genet* **45**∶191–196.

### SNP genotype-based Allelic Imbalance Analysis

**Figure 2:**
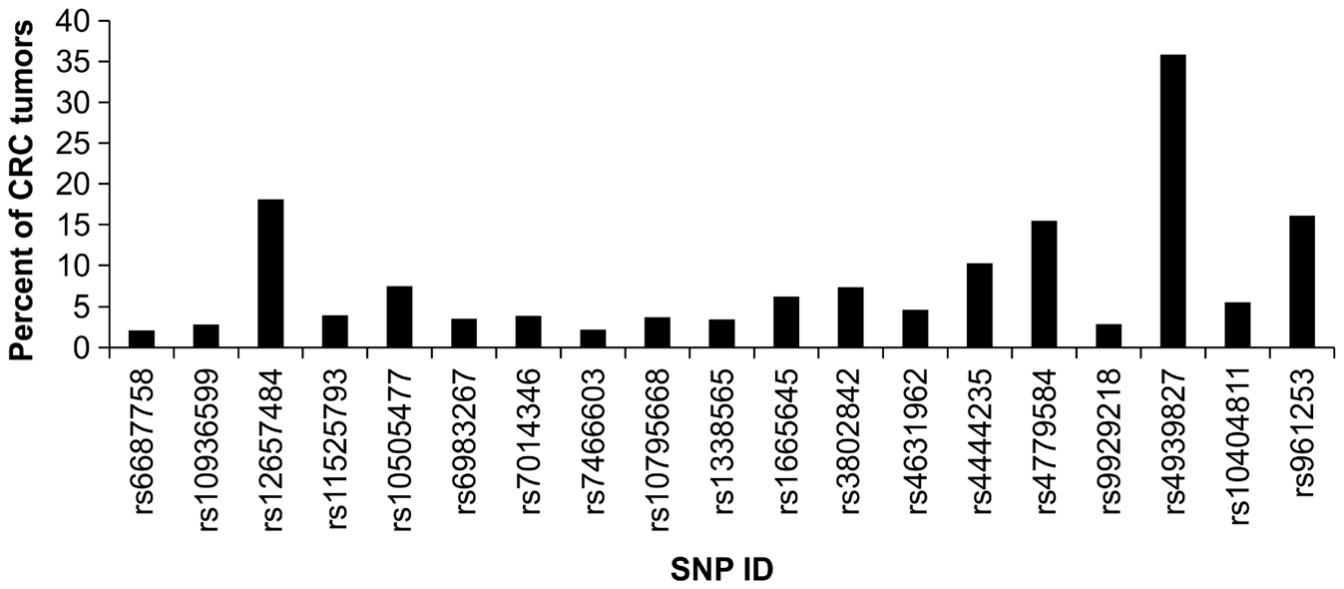
The percent of tumors with loss-of-heterozygosity compared to paired adjacent non-tumors. Fifteen reported and four newly-identified CRC-susceptibility SNPs were genotyped in tumor and adjacent non-tumor tissues in 705 CRC cases. Only cases with heterozygous genotypes, which provide di-allelic information, were used, and the total number of informative cases carrying homozygous calls in tumor tissues was measured as the LOH percentage.

**Figure 3:**
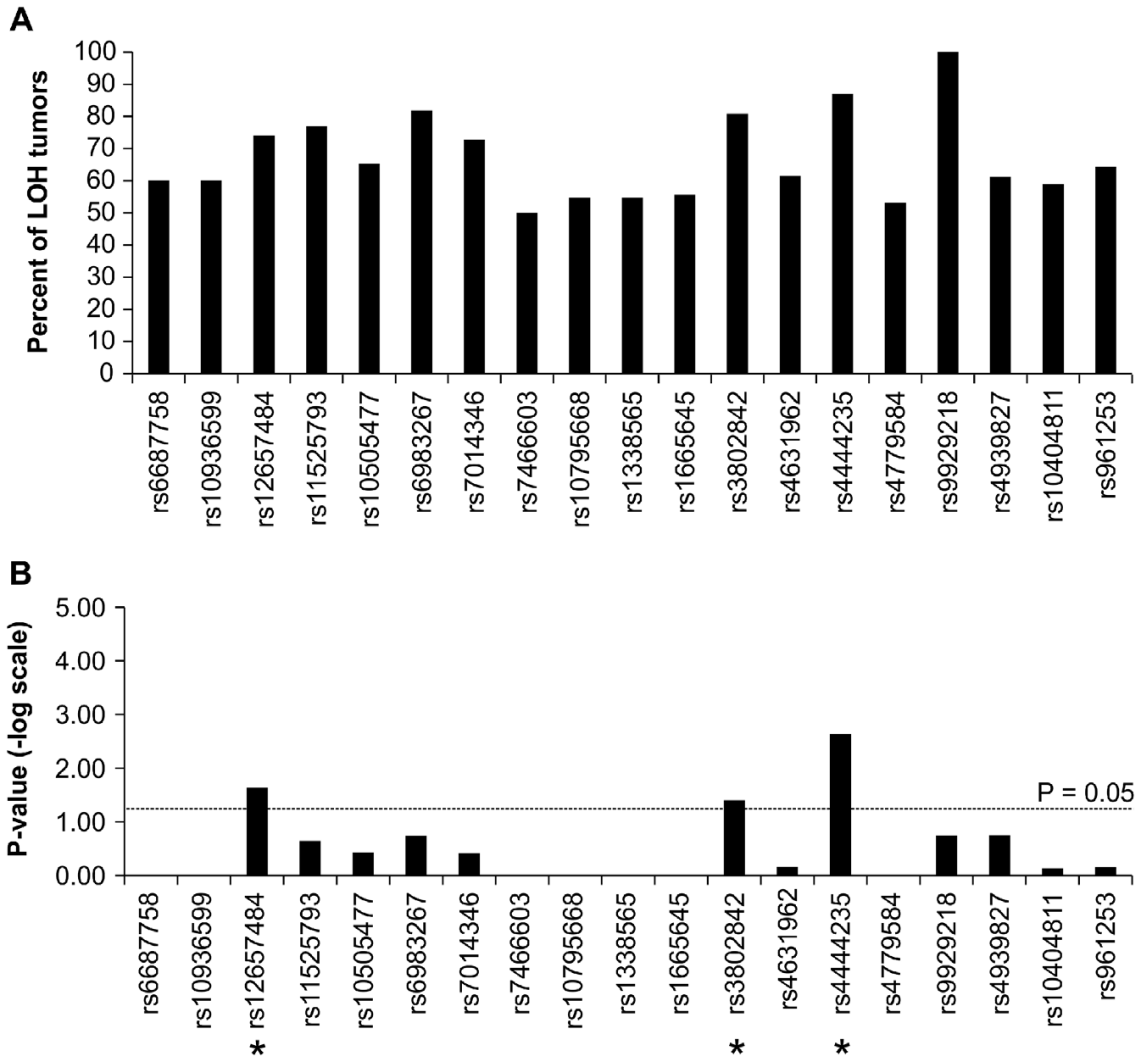
The predominance of risk allele in tumors. (A) The percent of tumors with risk allele retention. (B) Statistical significance of risk allele retentions. The difference in the retenion of specific alleles was compared using Fisher exact tests. Red dashed line indicates p = 0.05. The asterisk indicates significant p-value.

In order to detect allelic imbalance associated with neoplastic progression, these 19 SNPs were genotyped in the tumor tissues of the same 705 CRC patients. A conserved method was used to identify allele-specific imbalance events in these tumor samples. Since tumor tissues are highly heterogeneous, a simple and direct way to identify allelic imbalance is to apply well-established genotyping algorithm to call reliable and highly conserved SNP genotypes. Therefore, we applied the default algorithm to call the genotypes of 15 SNPs in both tumor and non-tumor tissues (Detail genotyping data are shown in Figure S2 in File S1). Only individuals carrying heterozygous genotype in non-tumor tissues provided information for the following analyses. First, we used the genotypes of each SNP of each paired samples to detect LOH events, and the LOH percent of each SNP ranged between 2 and 36% (Table 2). Because we used a conserved algorithm (Sequenom Typer algorithm) to call SNP genotype of tumor samples, there might be a bias towards copy number loss. Fifty tumors out of 276 (18%) associated with heterozygous normal tissue showed LOH in rs12657484 (Table 2). LOH in rs3802842 and rs4444235 occurred in only 7% and 10% of tumors (Figure 2). Furthermore, we measured the major-allele retention of each SNP as shown in Figure 3A. Among the SNPs, we observed some specific alleles of SNPs were unequally retained in tumor tissues. Significant retention of specific allele imbalance of rs4444235 was found in tumor (Bonferroni –adjusted p-value = 0.0437; unadjusted p-value = 0.0023) as shown in Figure. 3B.

**Table 2:** SNP loss-of-heterozygosity (LOH) analysis of tumor and adjacent non-tumor samples in colorectal cancer.

rs#	Chr	Position	No of adjacentnon-tumors with heterozygousgenotype	No of tumors with Allele1 homozygous genotype	No of tumors with Allele2 homozygous genotype	Total No of tumors with LOH	The percent of tumors with LOH	The percent ofMajor-GenotypeShift	Fisher Exact Test unadjusted p-value
rs6687758	1	222164948	237	2	3	5	2%	60%	1
rs10936599	3	169492101	354	6	4	10	3%	60%	1
*rs12657484	5	134503751	276	37	13	50	18%	74%	0.0228
rs11525793	7	15648935	330	10	3	13	4%	77%	0.2262
rs10505477	8	128407443	308	8	15	23	7%	65%	0.3726
rs6983267	8	128413305	311	9	2	11	4%	82%	0.1827
rs7014346	8	128424792	284	8	3	11	4%	73%	0.387
rs7466603	9	94483198	92	1	1	2	2%	50%	1
rs10795668	10	8701219	297	5	6	11	4%	55%	1
rs1338565	10	44059676	320	5	6	11	3%	55%	1
rs1665645	10	118487954	292	10	8	18	6%	56%	1
*rs3802842	11	111171709	355	21	5	26	7%	81%	0.0399
rs4631962	12	4373132	281	8	5	13	5%	62%	0.6951
*rs4444235	14	54410919	302	27	4	31	10%	87%	0.0023
rs4779584	15	32994756	207	15	17	32	15%	53%	1
rs9929218	16	68820946	211	6	0	6	3%	100%	0.1818
rs4939827	18	46453463	251	55	35	90	36%	61%	0.1769
rs10404811	19	58154807	311	7	10	17	5%	59%	0.7319
rs961253	20	6404281	87	5	9	14	16%	64%	0.7036

Note: Fifteen reported and four newly identified CRC-susceptibility SNPs were genotyped in tumor and adjacent non-tumor tissues in 705 CRC cases. Only cases with heterozygous genotypes, which provide di-allelic information, were used in the following analysis. Differences in retention of specific alleles were compared using Fisher exact tests.

The asterisk indicates significant p-value.

## Discussion

The incidence of CRC has been rapidly increasing in the past few decades in developing Asian countries. European GWAS of CRC susceptibility have been replicated in the eastern Asian populations of Japan [30]–[32], Singapore [33], Hong Kong [34], and China [35]. The Bureau of Health Promotion reported that CRC was the most common malignancy in Taiwan, with an age-standardized incidence of 37.1 per 100,000 people in 2007 [4], [36]; in comparison, the same year saw a rate of approximately 45 cases per 100,000 people in the USA [37].

Many SNPs have been identified as high risk or low risk variations associated with CRC [38]–[40]. However, variations between ethnicities and regions, genetic variations (e.g., LD and allele frequency), and environmental factors (e.g., smoking, alcohol, and dietary patterns) have made it difficult to identify common CRC susceptibility loci [41], [42].

In this study, we verified that rs10795668 and rs4631962, previously identified as CRC susceptibility loci in an East Asian GWAS, are also associated with CRC risk in the Taiwanese population. A genome-wide screen of Taiwan CRC and normal samples produced a candidate CRC susceptibility locus, rs1338565.

rs10795668 was reported to be associated with CRC risk and conferred better overall survival [34], [35], [43]–[45]. However, GWAS and several replication studies found no risk association of this variant in CRC [46]–[52]. In addition, rs10795668 allele frequencies differ between the European, Japanese, and African American populations [47]. rs10795668 maps to an 82-kb LD block (8.73–8.81 Mb) within 10p14 [53], but little is known about the function of the SNP and no known protein-coding genes are present in the surrounding 400 kb region. Like most risk variants identified by GWAS, rs10795668 also resides outside a gene-coding region; the nearest predicted genes are BC031880 and LOC389936 located 0.4 Mb and 0.7 Mb away, respectively. rs10795668 may increase expression of *ATP5C1*, which encodes the gamma subunit of the catalytic core (F1) of the mitochondrial ATP synthase [54]. The mitochondrial ATP synthase plays a central role in cellular respiration. The Warburg effect, the metabolic switch from respiration (in the mitochondria) to glycolysis (in the cytosol), commonly occurs in tumor cells [55]. Increased expression of *ATP5C1* associated with the A allele of rs10795668 would be consistent with maintaining the activities of ATP synthase and cellular respiration and potentially inhibiting tumor progression in colorectal cancer.

The new CRC susceptibility locus rs10774214, distally located 150 kb upstream of *CCND2*, was identified in East Asians by GWAS [24]. It showed strong LD (r^2^ = 0.825) with rs4631962, for which the major allele frequency is known for healthy individuals in the Taiwan Biobank [56]. rs4631962 is proximally located just 10 kb upstream of *CCND2*, which encodes cyclin D2, a member of the D-type cyclin family. *CCND2* is a critical mediator of cell cycle control (from G1 to S phase) and is overexpressed in a substantial proportion of human colorectal tumors [57]–[60]. Overexpression of *CCND2* is an independent predictor of survival in individuals with CRC [59]. *PARP11*, *C12orf5*, *FGF6*, and *RAD51AP1*are also in close proximity to the SNP; *C12orf5* and *RAD51AP1* are overexpressed in CRC tissue [60]. rs461962 is in strong LD with several SNPs in potential transcription factor-binding sites in the TRANSFAC database [60].

The newly identified rs1338565 is located in an intron of the *ZNF239* gene on chromosome 10q11.22. Two neighboring SNPs, rs2230660 and rs2230661, produce missense variations in the exon region of *ZNF239* and are in strong LD with rs1338565. The biological rationale for the association between *ZNF239* and CRC has not been explored. *ZNF239* is a zinc-finger protein that recognizes both DNA and RNA. This dual affinity suggests *ZNF239* is involved in transcription and post-transcriptional regulation. As a DNA-binding transcriptional repressor, it represses the *IRBP* (interphotoreceptor retinoid-binding protein) gene by competing with the *CRX* (cone-rod homeobox protein) transcriptional activator for DNA binding [61]. Previous studies have shown *ZNF239* interactions with lamin A/C and the nuclear matrix may be important for its ability to repress transcription [62], [63]. Additional research may be warranted to understand the mechanisms by which this SNP is related to CRC risk.

CRC susceptibility-associated genetic variations have been found to affect gene expression through distant regulatory elements. Our study showed rs4939827 had the highest rate of LOH in CRC patients (36%). rs4939827 reduces *SMAD7* expression, leading to aberrant TGFβ signaling [64]; however, no significant difference in the alleles targeted by imbalance was detected in rs4939827 at 18q21 (p = 0.17) [64], [65]. Given that subtle changes in distant regulatory elements result in low-penetrance of cancer susceptibility and play a role in tumor cell development, alterations in several loci of *BMP4* at 14q22 and *GREM1* at 15q13 affect TGFβ signaling [43].

rs3802842 and rs4444235 have been associated with increased risk of CRC in different populations, but this association was not replicated in our study. rs12657484 showed strong LD (r^2^ = 0.926) with rs647161, which is a newly discovered CRC susceptibility locus in East Asians [24]. A recent study showed rs3802842 and rs4444235 have no allelic imbalance in the Finnish population [66]. However, our study demonstrated rs12657484, rs3802842, and rs4444235 exhibit LOH in the Taiwanese population.

Rs1265484 is located on chromosome 5q31.1, where a cluster of SNPs are associated with CRC risk [24]. Of the genes in this region (including *PITX1*, *CATSPER3*, *PCBD2*, *MIR4461*, and *H2AFY*), *PITX1* is closer to rs12657484 (approximately 134 kb upstream) than rs647161 (approximately 157 kb upstream). The *PITX1* gene (encoding paired-like homeodomain 1) has been reported as a tumor suppressor and may be involved in the tumorigenesis of multiple human cancers [67]–[70], including CRC [67], [71]. Several studies have demonstrated reduced expression of *PITX1* human cancer tissues and cell lines; *PITX1* suppresses tumorigenicity by down-regulating the RAS pathway [67]–[71]. Lower expression of *PITX1* was observed in KRAS wild-type CRC tissue, not *KRAS*-mutant tissue [71]. *PITX1* may also activate *TP53* [72] and regulate telomerase activity [73]. Low *PITX1* expression has been associated with poor survival in CRC patients [74].

The association of rs3802842 (11q23), located in the intron of *C11orf93*, with CRC risk was first identified in a GWAS [38]. Little is known about the function of rs3802842, but it may affect *C11orf93* expression by altering the transcription factor binding site [75]. It may also alter the expression of genes outside 11q23.1 through cis- or trans-regulatory mechanisms [76]. Within 100 kb of rs3802842 is a cluster of ORFs (*POU2AF1*, *C11orf53*, *FLJ45803*, and *LOC120376*) and a SNP (rs12296076) identified as polymorphic binding sites for miRNAs in high linkage disequilibrium [38]. The genes encoding POU transcription factors are near rs3802842 [38].

Previously published GWAS found a significant association between CRC susceptibility and rs4444235 located on chromosome 14q22.2 within the bone morphogenetic protein-4 (*BMP4*) gene [38], [42], [77]. rs4444235 is a cis-acting regulator of *BMP4* expression. Given that the overexpression of the BMP genes could suppress the Wnt signaling pathway by preventing β-catenin activation and increasing migration and invasion of the mesenchymal phenotype in CRC [78]–[80], controlling BMP signaling is critical to maintaining the Wnt signaling associated with CRC development [81].

In a recent study, 16 SNPs previously associated with CRC risk for allele-specific imbalance were investigated [82]. Only rs6983267 variant showed statistical significance of allele-specific imbalance from individual of European ancestry. But the SNP was not replicated in our study and it may be due to our tumor number of LOH limitation. The percent of major-genotype shift was high (82%) for this SNP, but perhaps because only 11 total tumors showed LOH.

In conclusion, several CRC-susceptibility SNPs identified in East Asian studies are also associated with increased CRC risk in the Taiwanese population. However, the incongruent results between this study and previous East Asian associations could be attributed to the racial and ethnic differences of the respective study populations because allele frequencies of these SNPs may be different. Another possible reason that these SNPs were not found to be susceptibility variants for CRC might be that the sample size was not large enough to provide sufficient statistical power in this study. Despite the small sample size of this replication study, the GWAS-identified novel SNP rs1338565 was associated with an increased risk of CRC in our population. The East Asian CRC-susceptibility SNPs rs10795668 and rs46310962 also contribute to the risk of CRC in Taiwan. We investigated the 19 low-penetrance CRC loci and identified three loci, rs12657484, rs3802842, and rs4444235, exhibiting LOH in tumor tissue compared to matched adjacent heterozygous normal tissue. The LOH reveals whether a variant acts as a tumor suppressor or an oncogene, and guides further functional studies.

## Supporting Information

File S1 **Supporting information file containing Figures S1 and S2; Table S1. Figure S1:** Principal component analysis of cases and controls using unlinked SNPs. To clarify the possibility of population stratification, 44 widely used unlinked SNPs were used for genotyping non-tumor tissues of 705 cases, and then combined with the corresponding SNP data of 1,802 controls from Taiwan Biobank database for principal component analysis. The result indicated that there is no obvious population stratification within all samples. **Figure S2:** The sequenom cluster of tumor and non-tumor data. Nineteen SNPs that were typed in 705 independent CRC pairs of tumor (T) and paired normal adjacent tissues (N) using Sequenom iPLEX genotyping method and default calling algorithm. **Table S1:** Allele frequency comparisons of 705 cases and 1,802 controls using 44 unlinked SNPs.
(DOCX)
