# Supplementary material for: Single Nucleotide Polymorphisms Associated with Colorectal Cancer Susceptibility and Loss of Heterozygosity in a Taiwanese Population
Source: PLoS One. 2014 Jun 26;9(6):e100060. doi: 10.1371/journal.pone.0100060 (PMC4072675; doi:10.1371/journal.pone.0100060)
Supplement: File S1 — Supporting information file containing Figures S1 and S2; Table S1. Figure S1: Principal component analysis of cases and controls using unlinked SNPs. To clarify the possibility of population stratification, 44 widely used unlinked SNPs were used for genotyping non-tumor tissues of 705 cases, and then combined with the corresponding SNP data of 1,802 controls from Taiwan Biobank database for principal component analysis. The result indicated that there is no obvious population stratification within all samples. Figure S2: The sequenom cluster of tumor and non-tumor data. Nineteen SNPs that were typed in 705 independent CRC pairs of tumor (T) and paired normal adjacent tissues (N) using Sequenom iPLEX genotyping method and default calling algorithm. Table S1: Allele frequency comparisons of 705 cases and 1,802 controls using 44 unlinked SNPs. (DOCX) [file pone.0100060.s001.docx]

**Supporting Information**


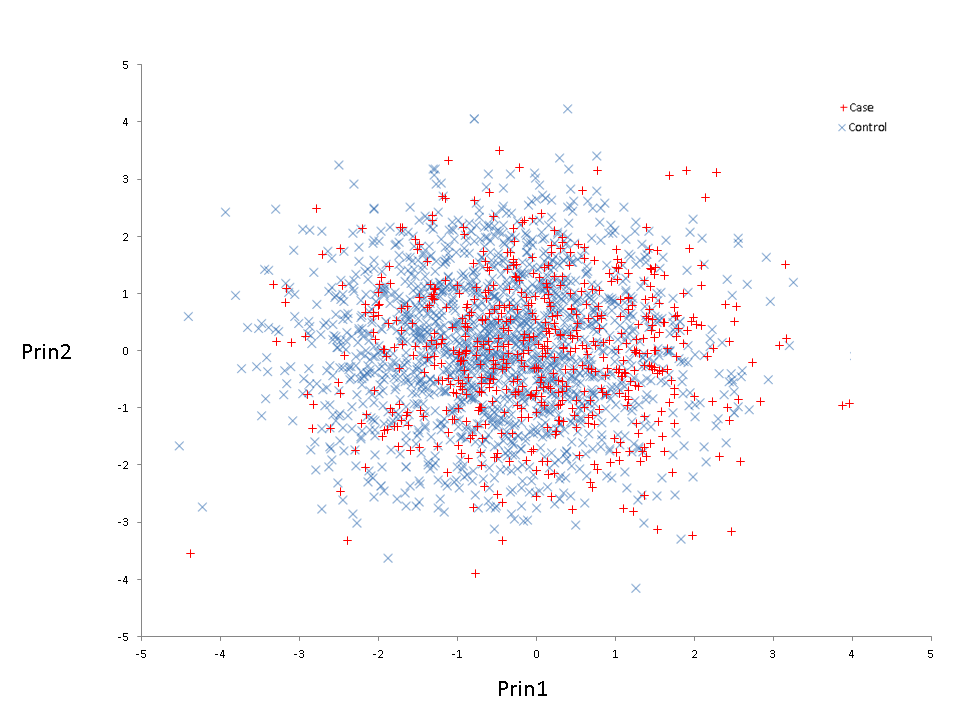


**Figure S1 Principal component analysis of cases and controls using unlinked SNPs.** To clarify the possibility of population stratification, 44 widely-used unlinked SNPs were used to genotype in non-tumor tissues of 705 cases, and then combined the corresponding SNP data of 1,802 controls from Taiwan Biobank database for principal component analysis. The result indicated that there is no obvious population stratification within all samples.


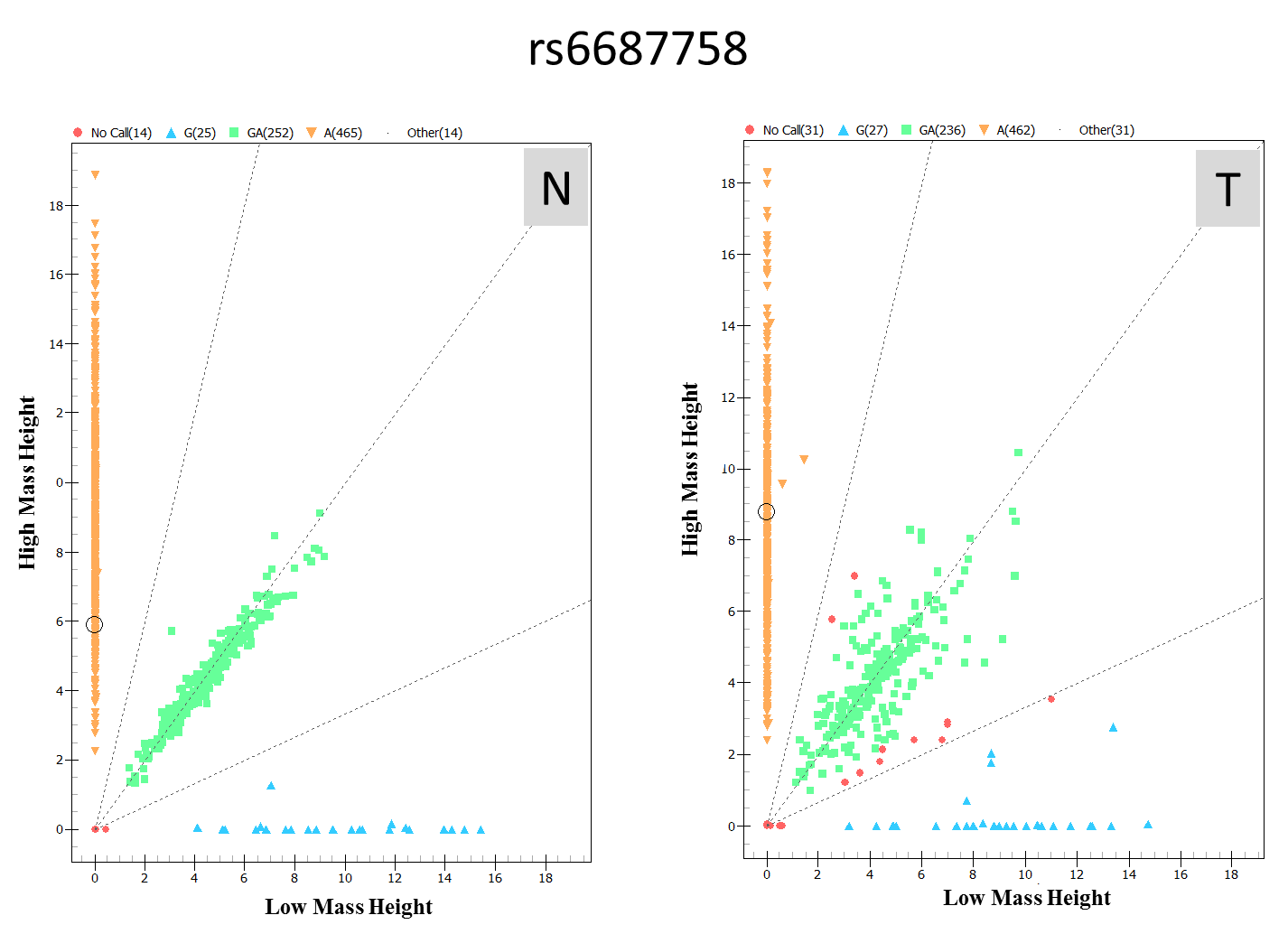


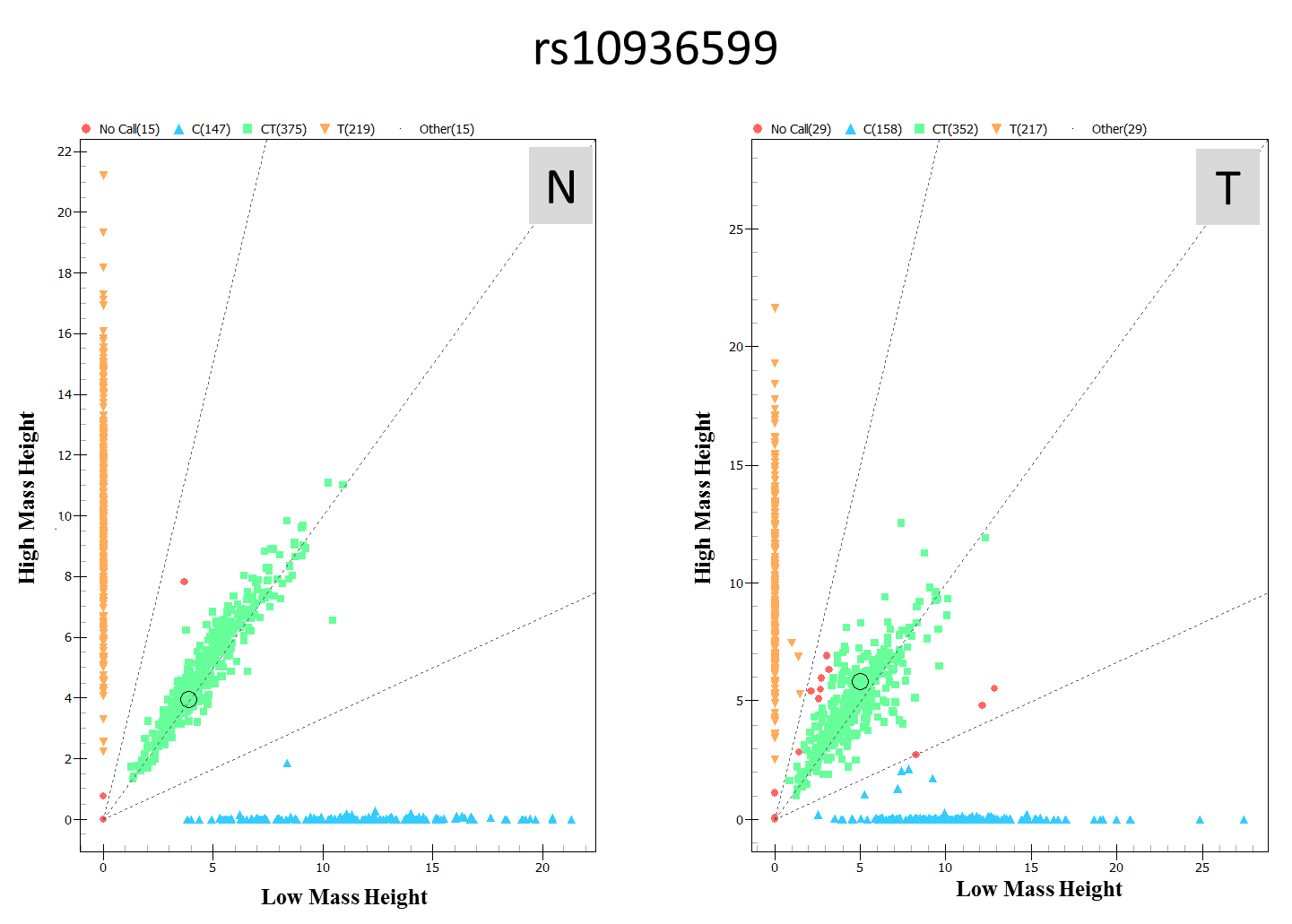


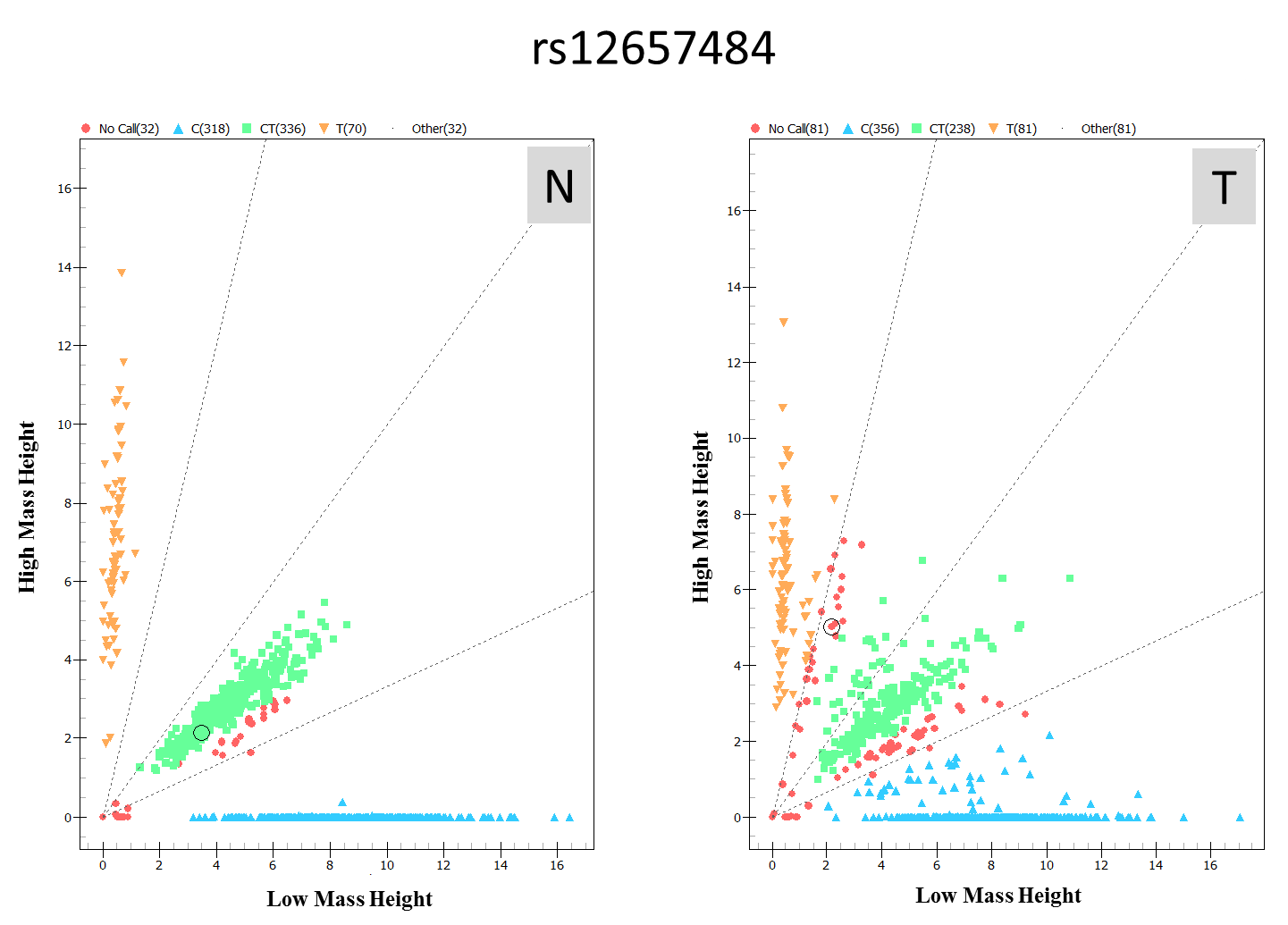


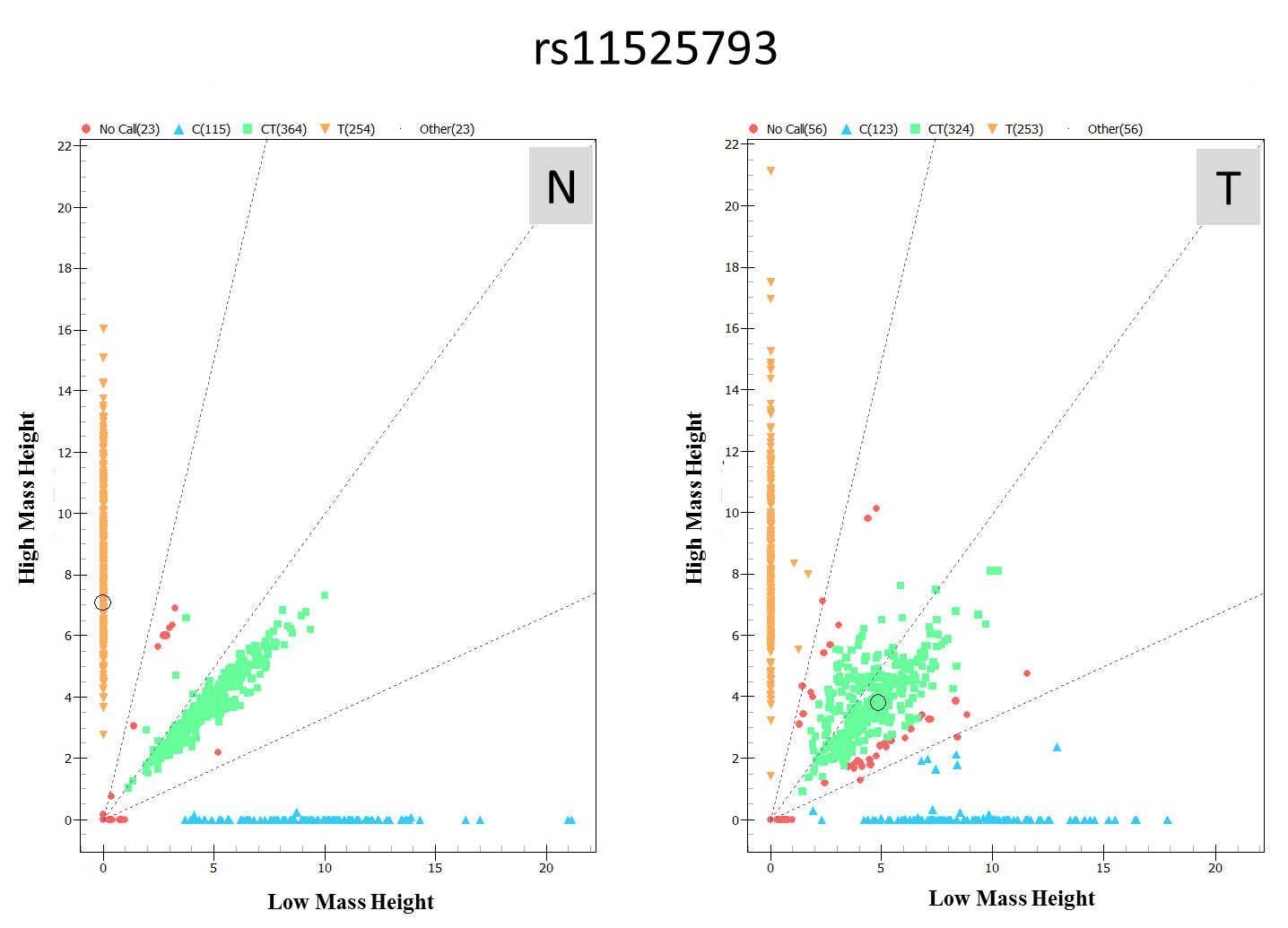


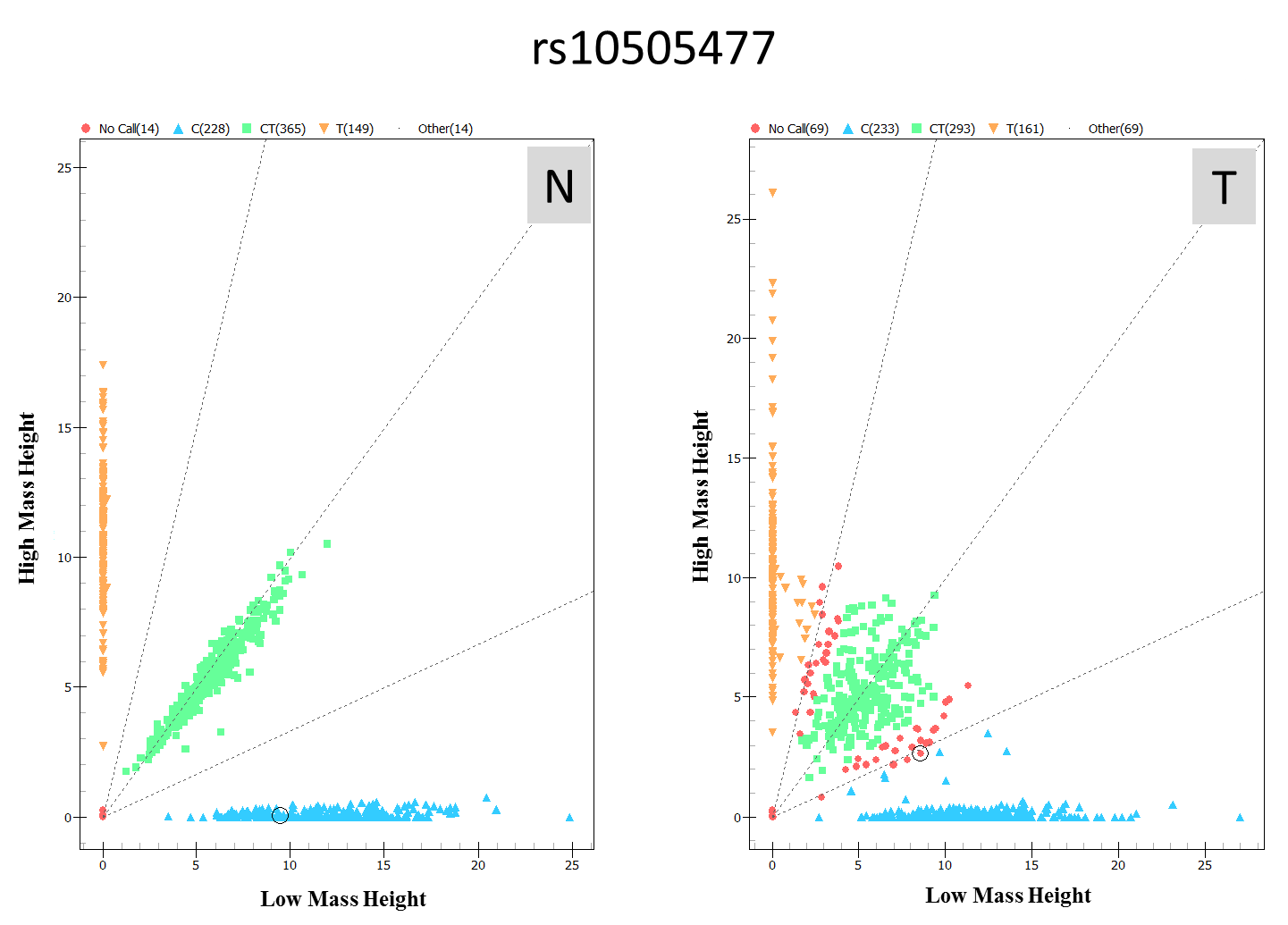


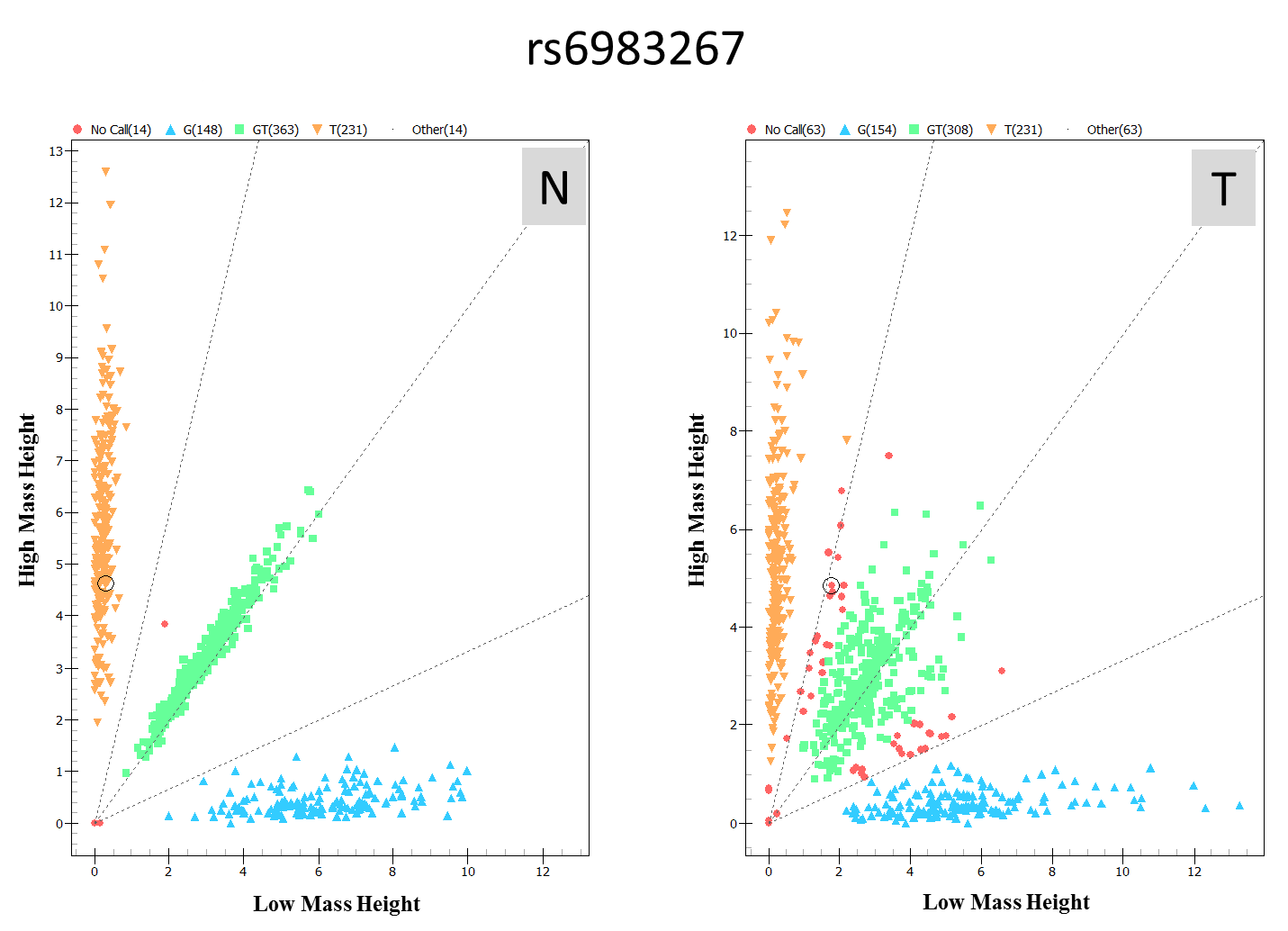


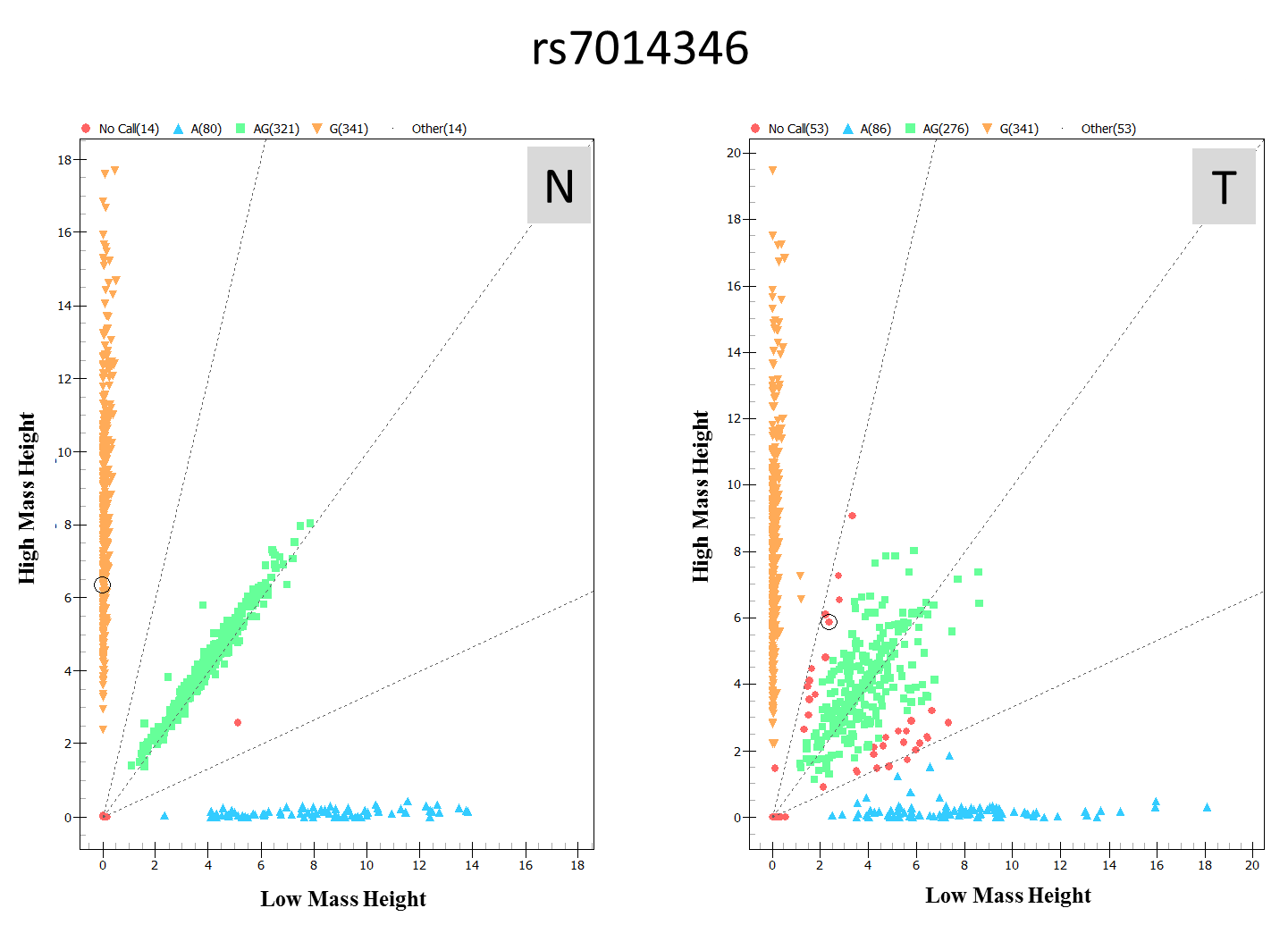


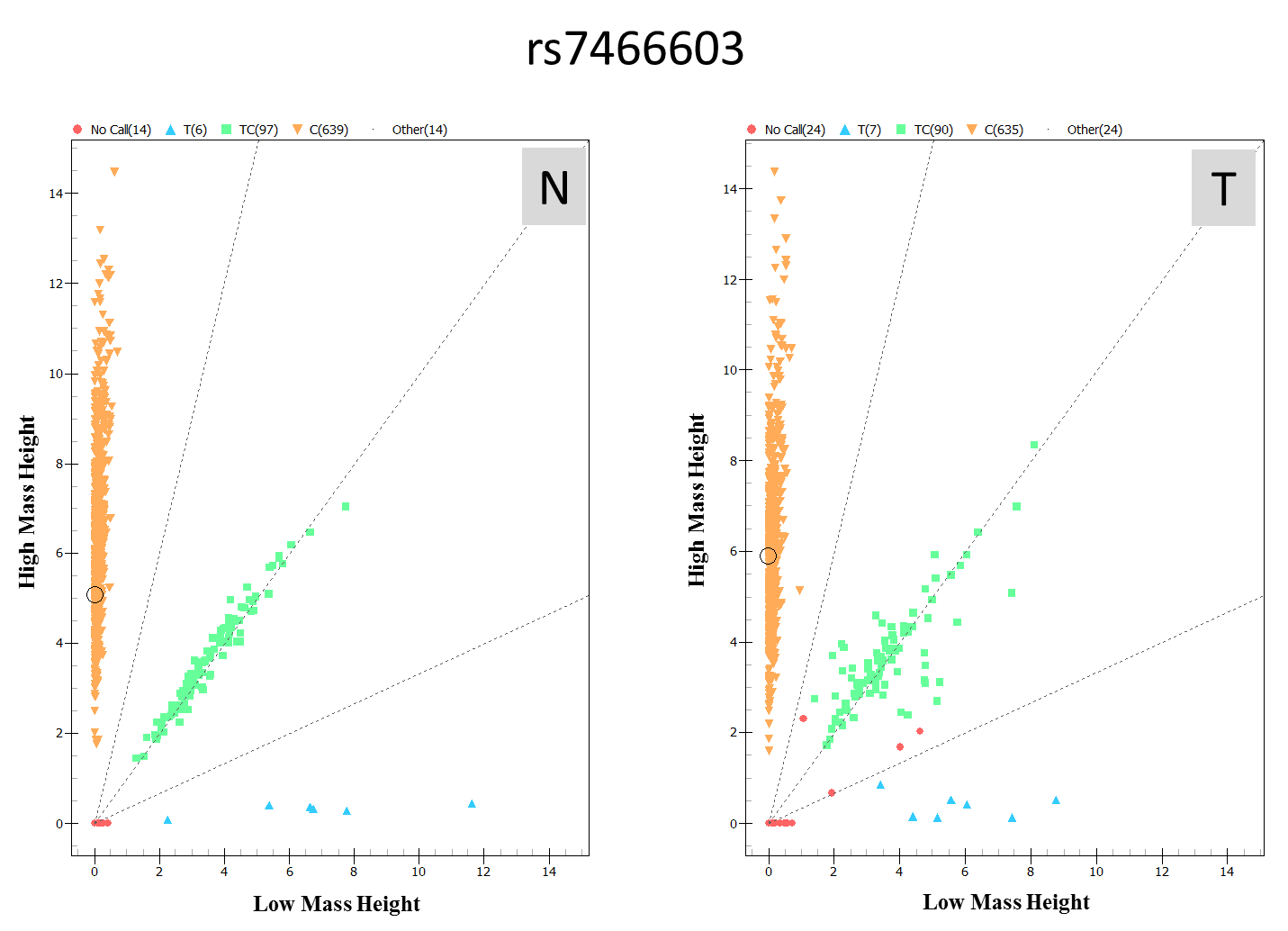


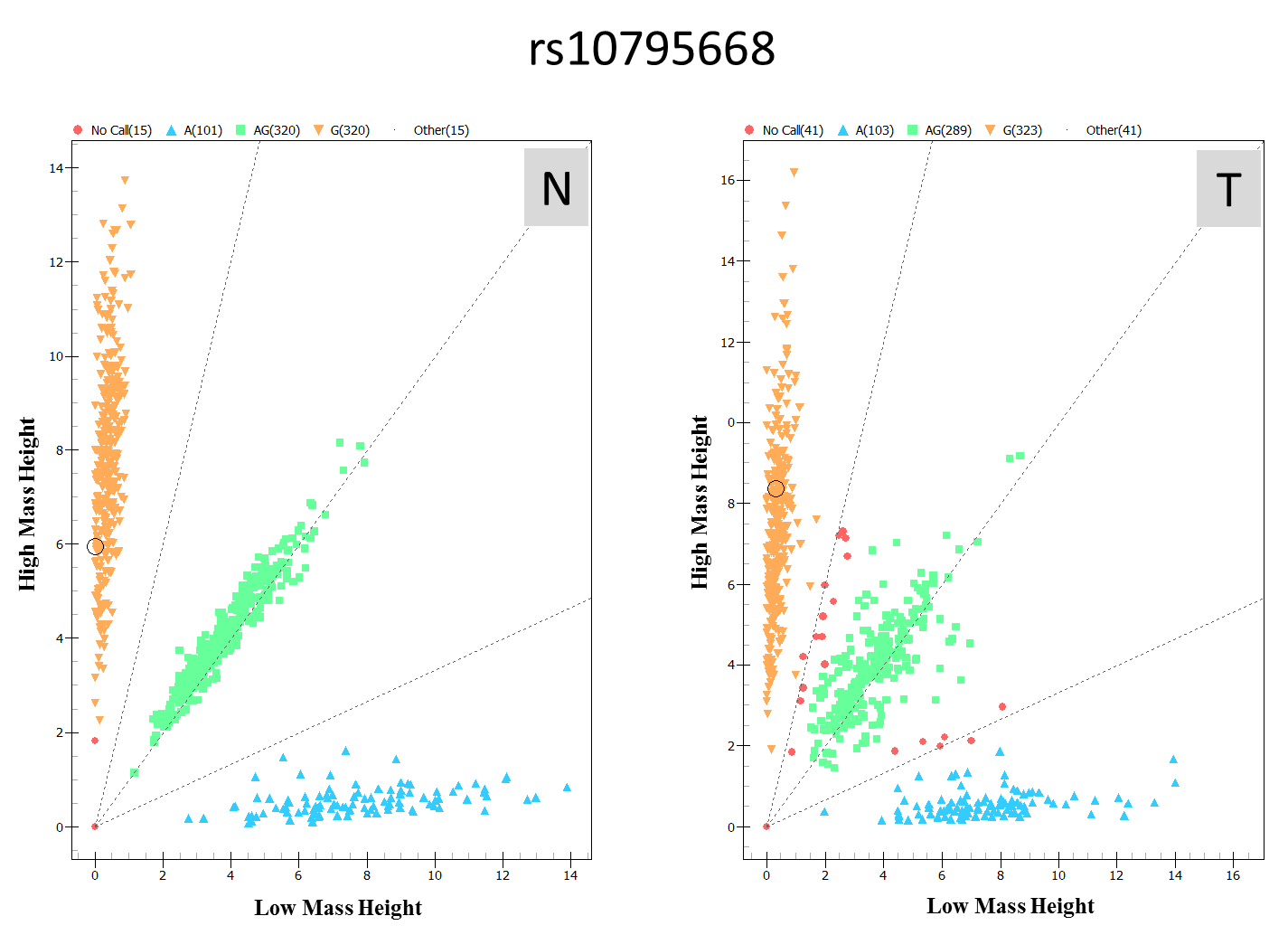


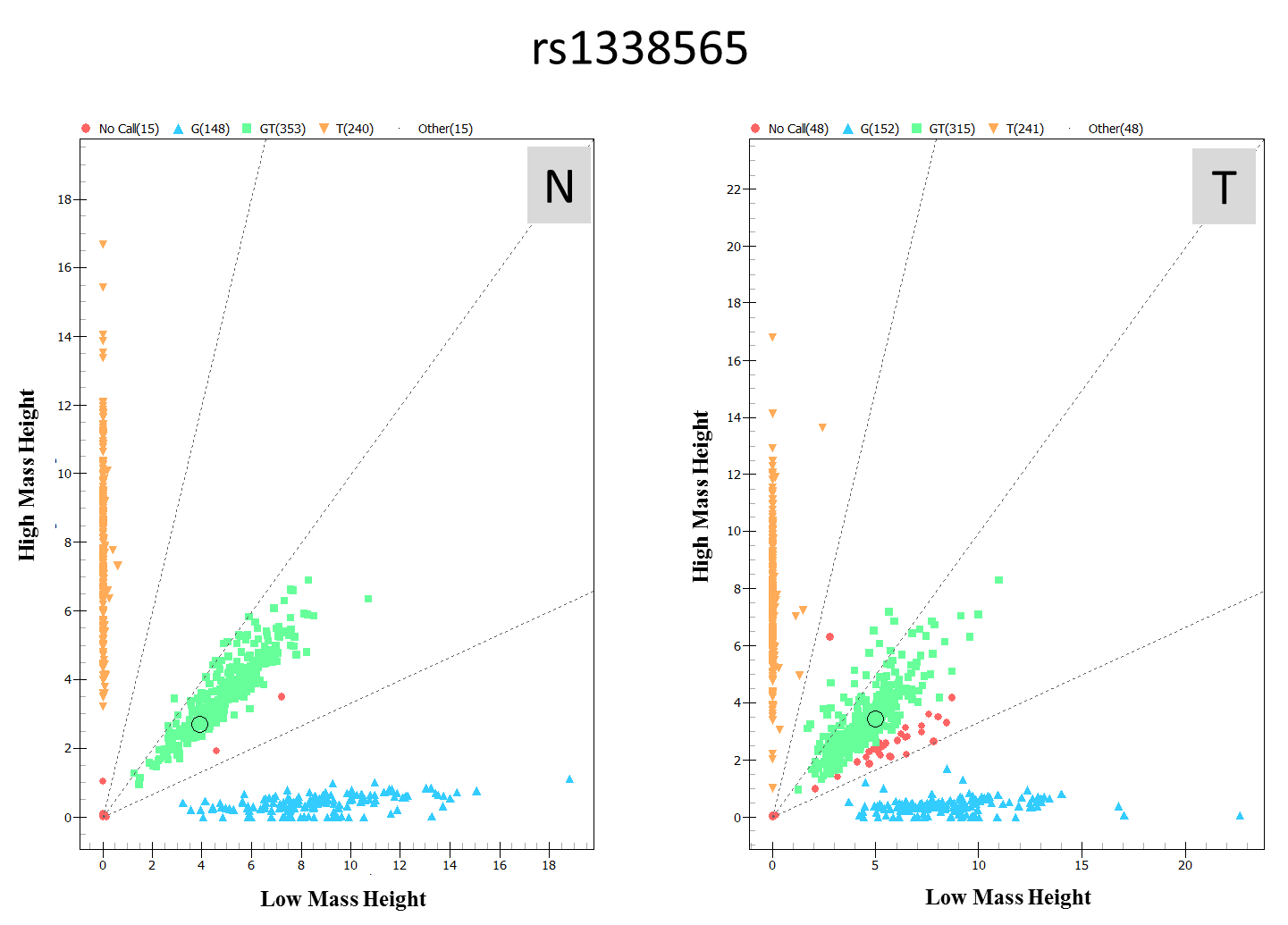


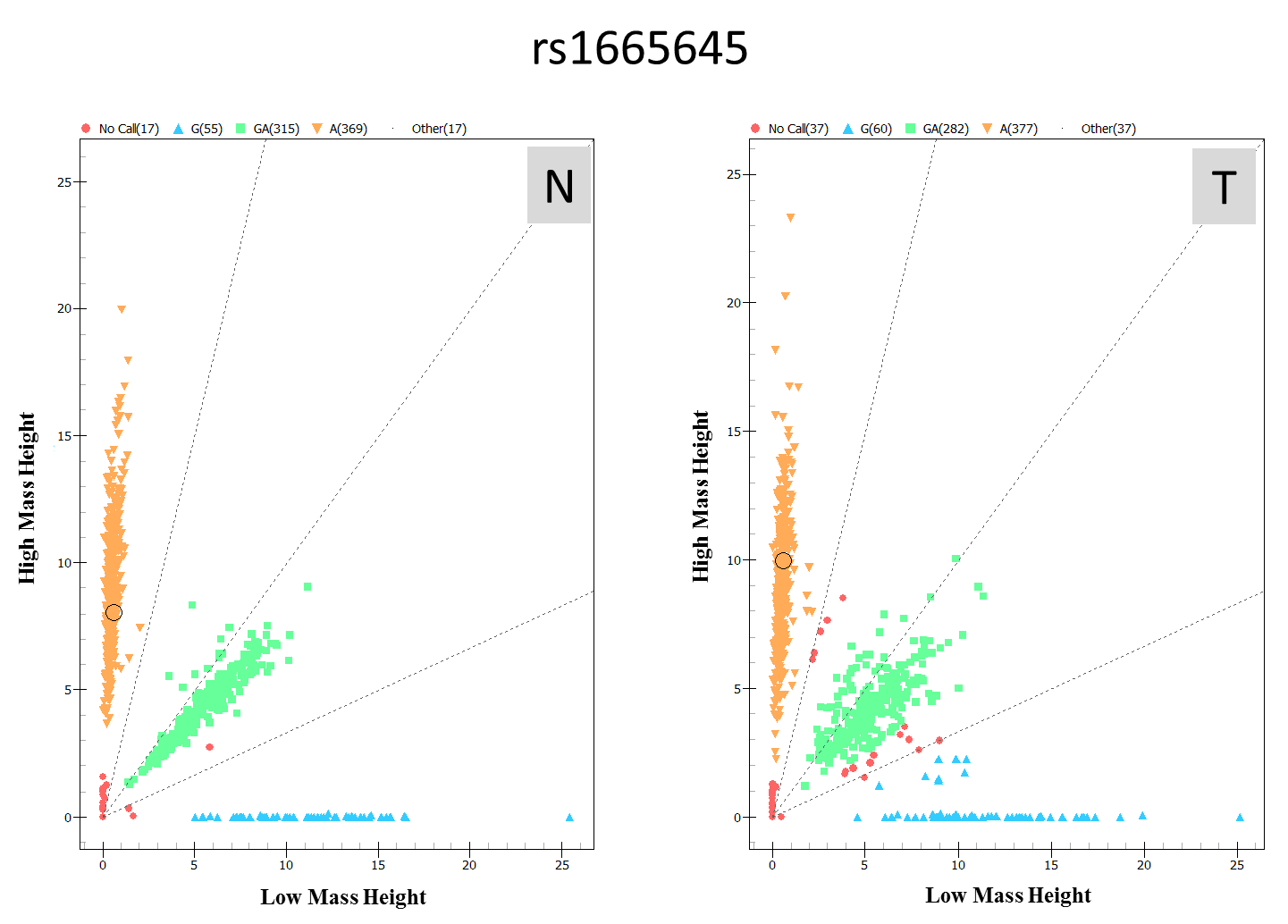


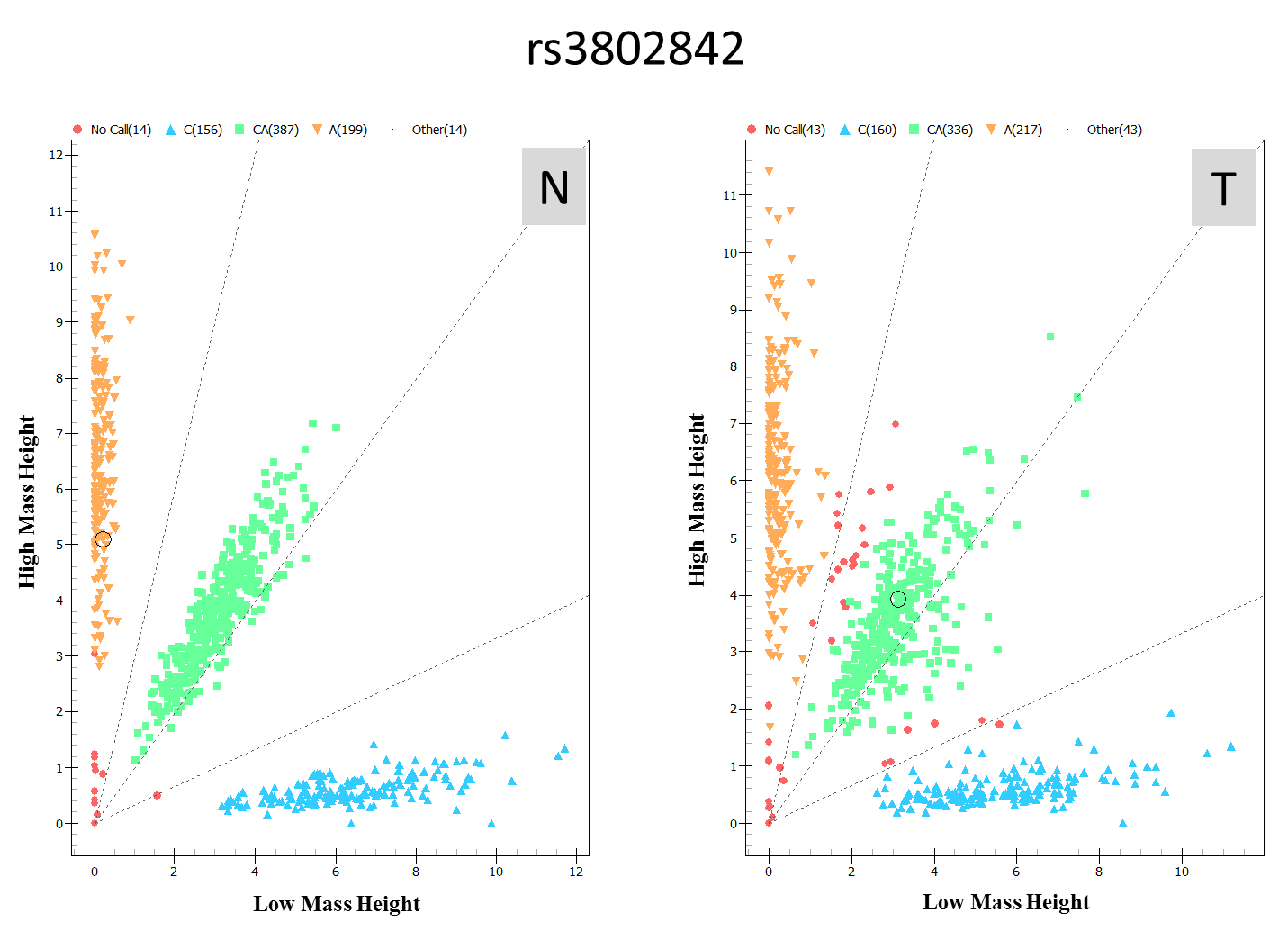


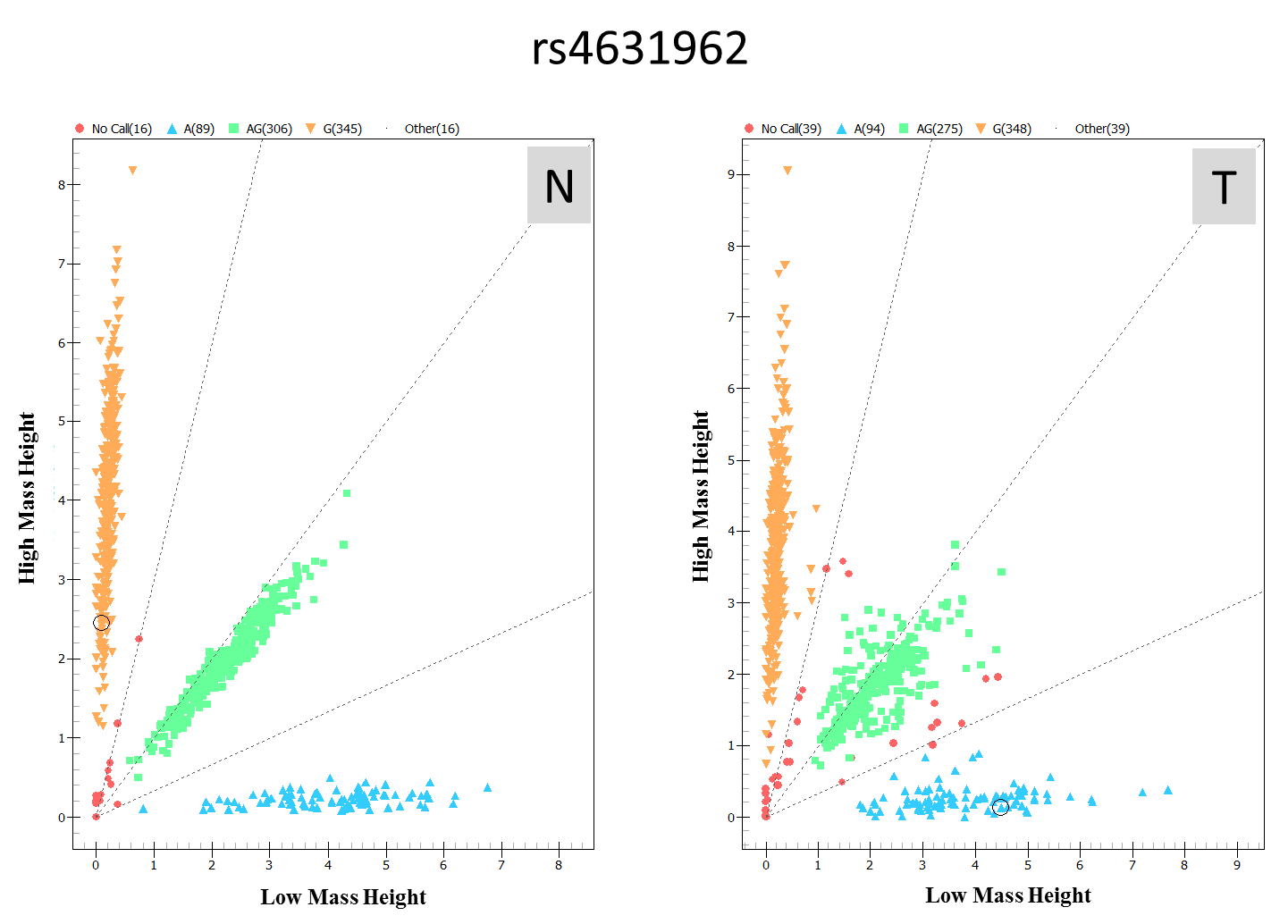


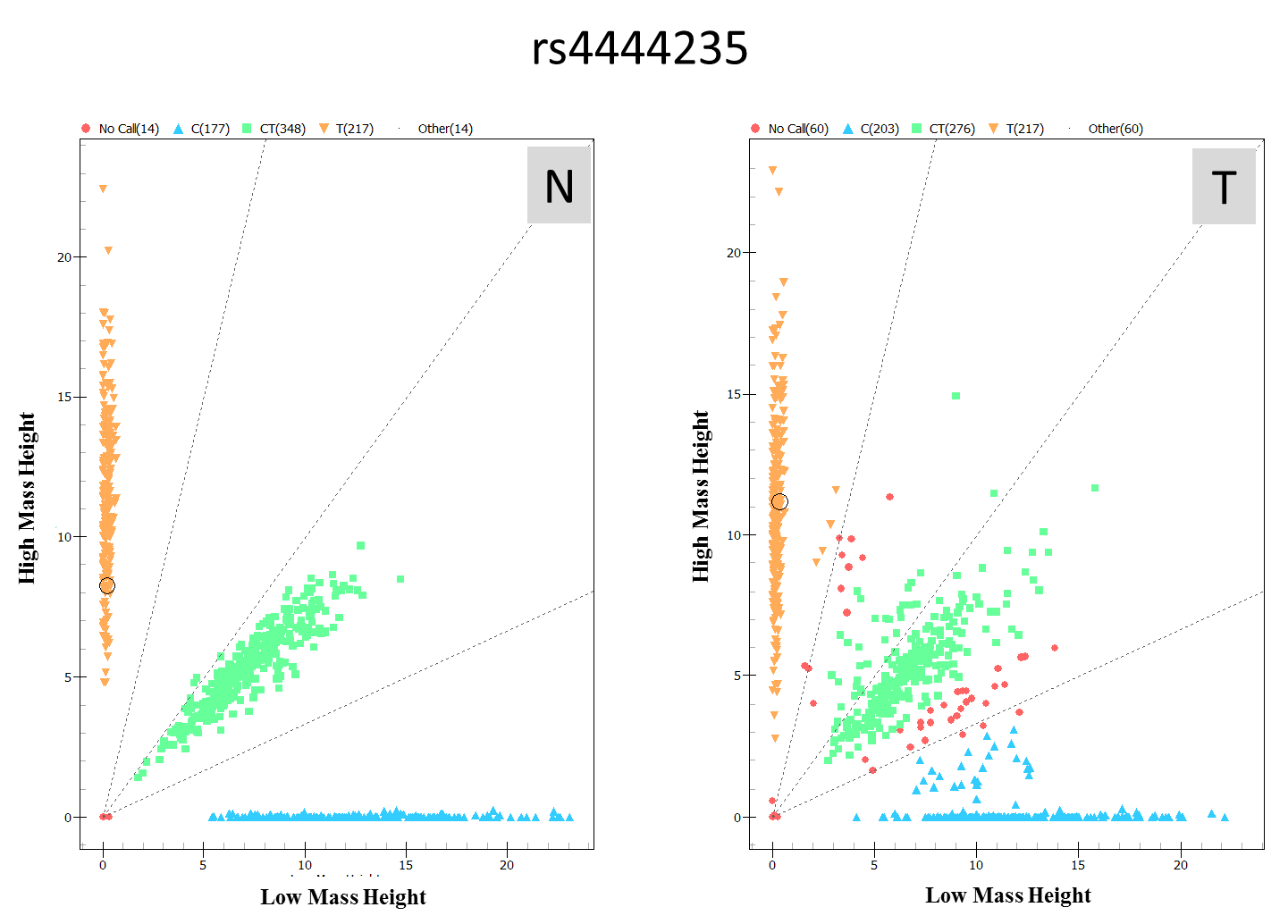


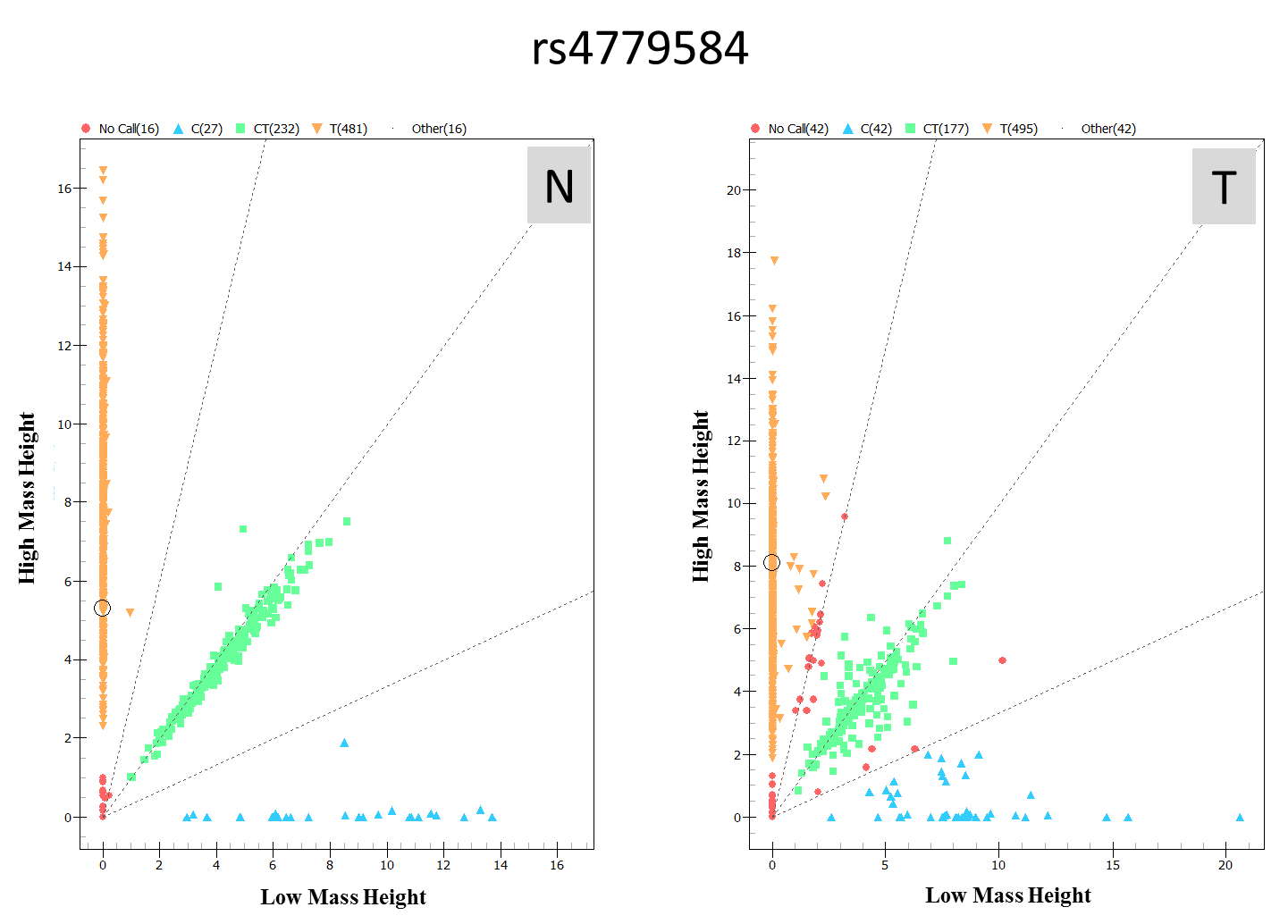


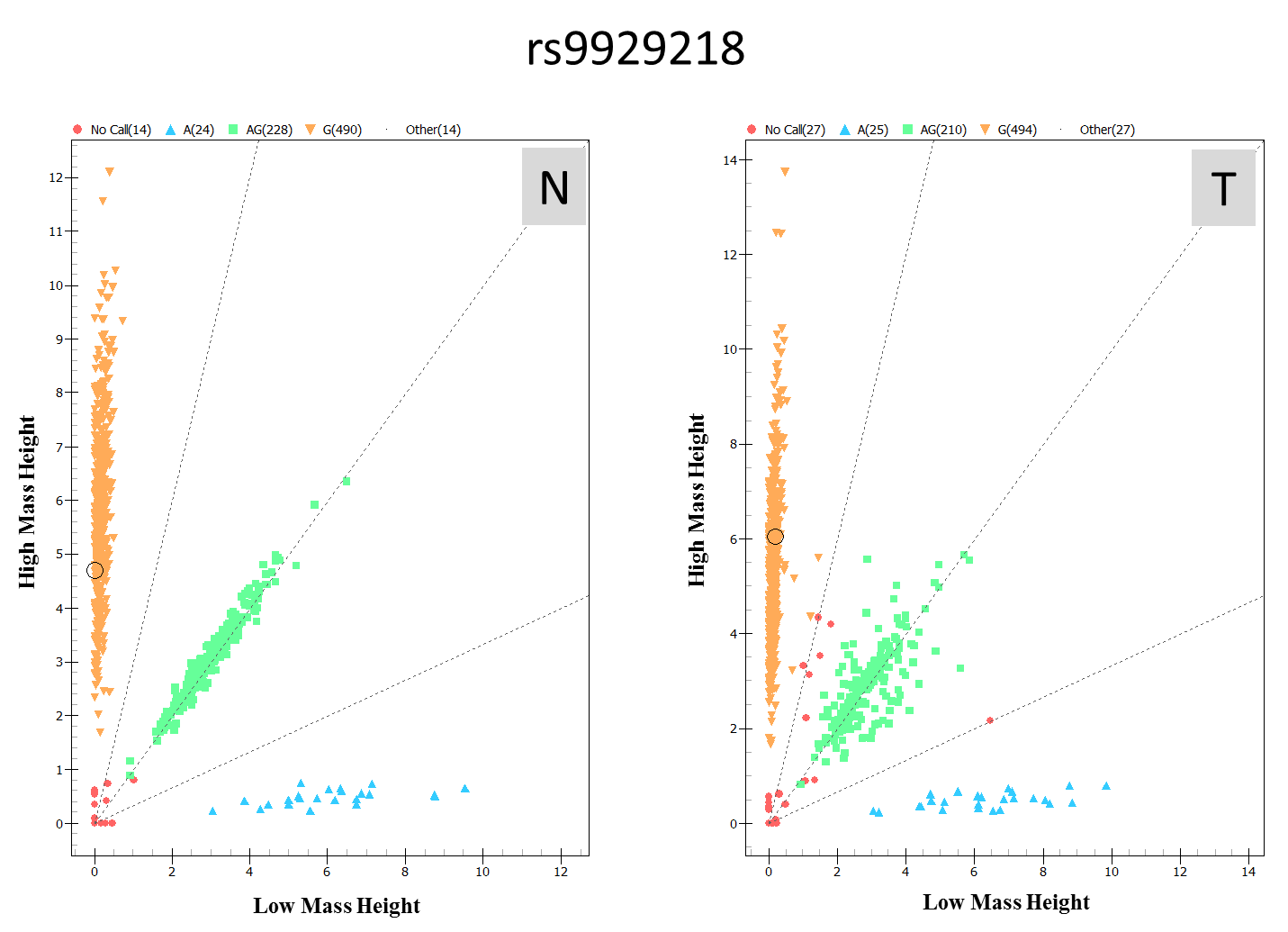


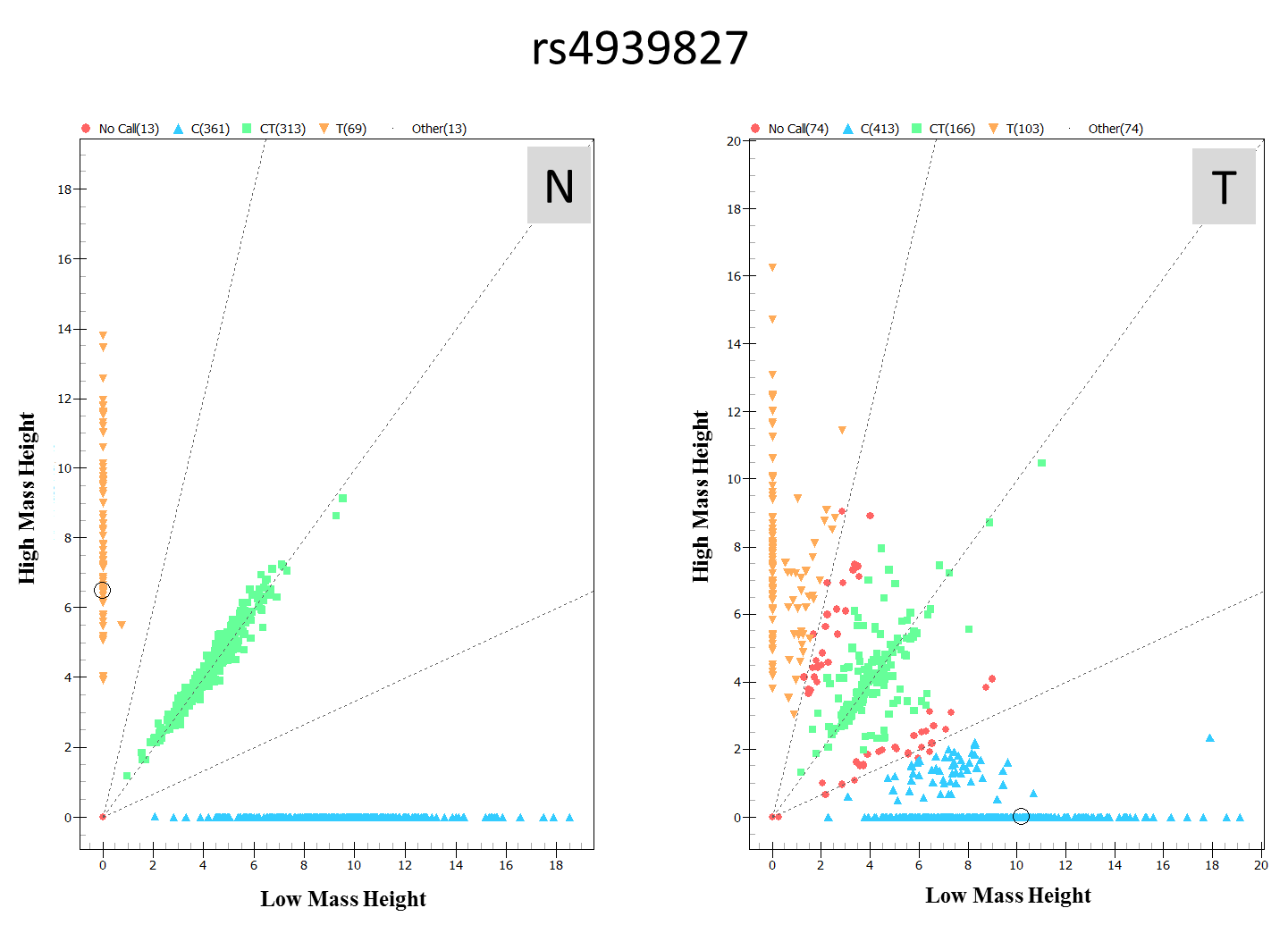


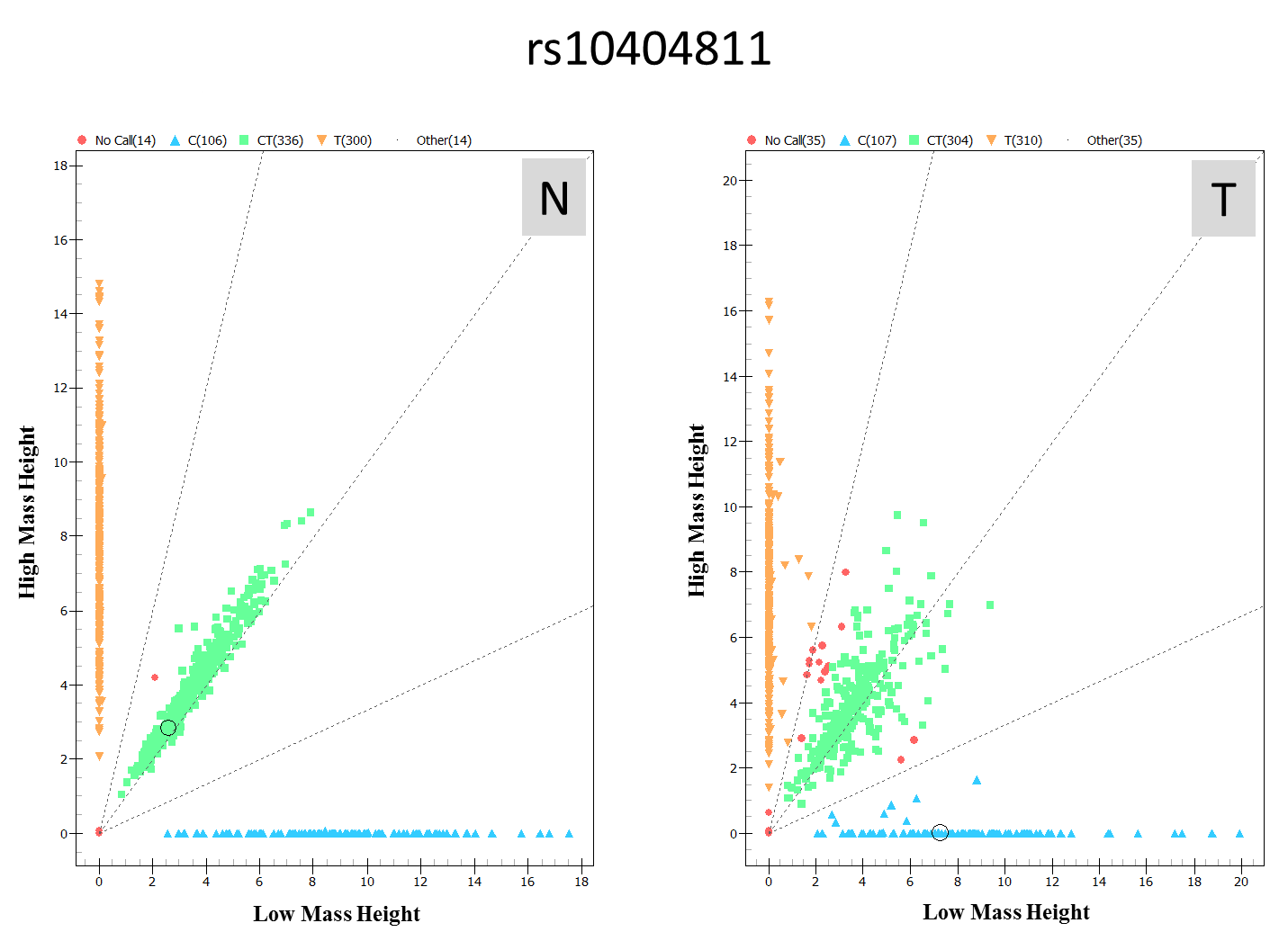


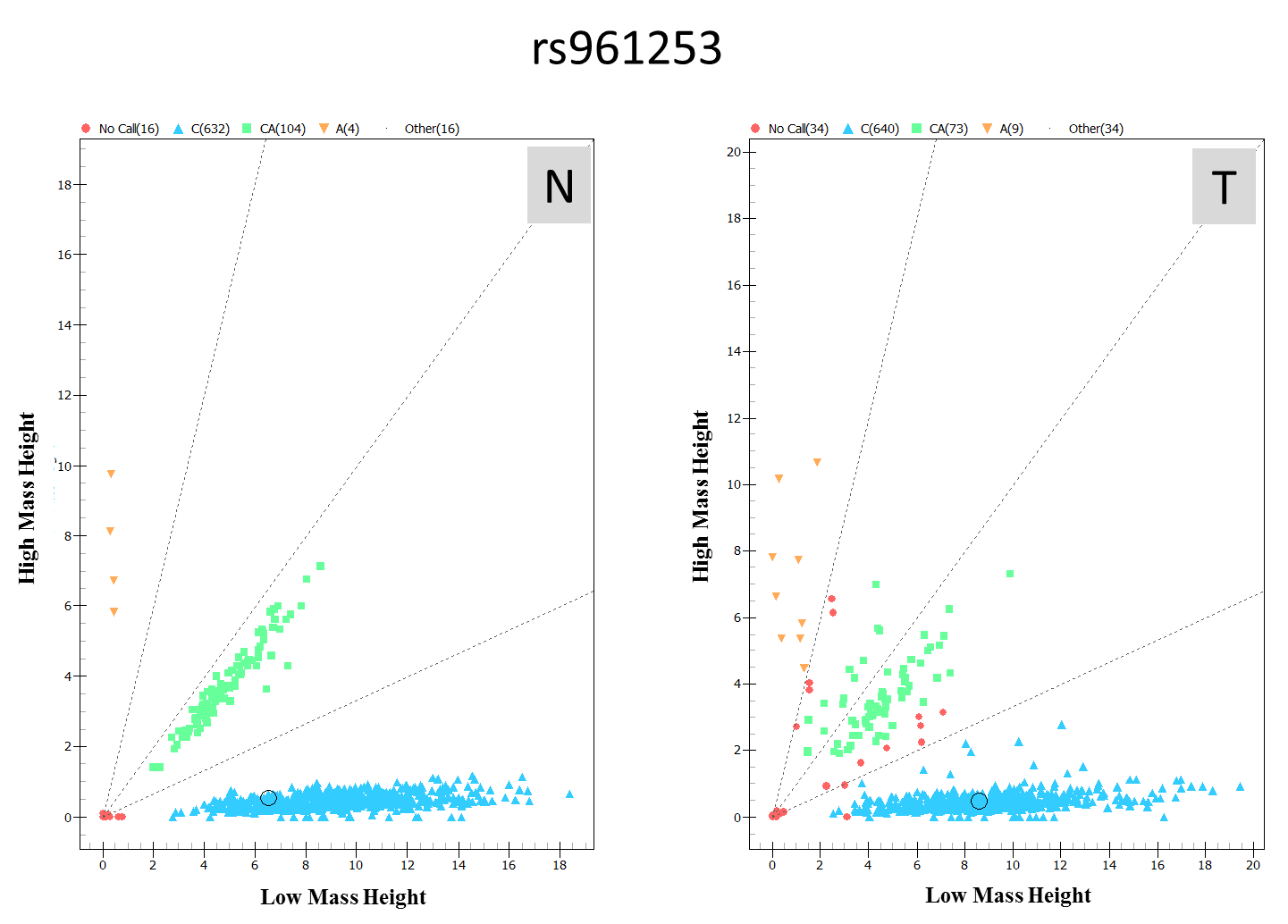


**Figure S2. The Sequenom cluster figures of tumor and non-tumor data**.

Nineteen SNPs were typed in 705 independent CRC pairs of tumor (T) and paired normal adjacent tissues (N) using Sequenom iPLEX genotyping method and default calling algorithm.

| **Table S1. Allele frequency comparisons of 705 cases and 1,802 controls using 44 unlinked SNPs** | | | | | | | | |
| --- | --- | --- | --- | --- | --- | --- | --- | --- |
| **Chromosome** | **Position** | **rs#** | **Minor Allele** | **Major Allele** | **Case MAF** | **Control MAF** | **HW Test (p-value)** | **Allele chi-square test unadjusted p-value** |
| 1 | 220,145,584 | rs1982986 | A | G | 0.364 | 0.371 | 0.751 | 0.614 |
| 1 | 238,439,308 | rs10495407 | A | G | 0.295 | 0.298 | 0.114 | 0.790 |
| 1 | 239,881,926 | rs891700 | G | A | 0.440 | 0.451 | 0.775 | 0.405 |
| 1 | 242,806,797 | rs1413212 | C | T | 0.488 | 0.499 | 0.239 | 0.389 |
| 2 | 114,974 | rs876724 | T | C | 0.395 | 0.400 | 0.731 | 0.723 |
| 3 | 961,782 | rs1357617 | A | T | 0.212 | 0.222 | 1.000 | 0.382 |
| 3 | 186,032,934 | rs3819854 | A | G | 0.340 | 0.347 | 0.264 | 0.533 |
| 4 | 10,969,059 | rs2046361 | A | T | 0.432 | 0.428 | 0.716 | 0.800 |
| 5 | 2,879,395 | rs717302 | G | A | 0.113 | 0.119 | 0.271 | 0.522 |
| 5 | 174,778,678 | rs251934 | G | A | 0.129 | 0.143 | 0.291 | 0.123 |
| 6 | 2,559,898 | rs214054 | A | G | 0.377 | 0.385 | 0.334 | 0.549 |
| 6 | 165,045,334 | rs727811 | G | T | 0.369 | 0.358 | 0.433 | 0.423 |
| 7 | 17,955,294 | rs2714854 | C | T | 0.404 | 0.425 | 0.562 | 0.133 |
| 8 | 40,554,742 | rs11781516 | C | T | 0.441 | 0.440 | 0.886 | 0.931 |
| 8 | 139,399,116 | rs2056277 | T | C | 0.127 | 0.125 | 0.031 | 0.827 |
| 9 | 126,881,448 | rs1463729 | T | C | 0.457 | 0.464 | 0.048 | 0.586 |
| 9 | 128,968,063 | rs1360288 | T | C | 0.382 | 0.383 | 0.331 | 0.919 |
| 10 | 2,406,631 | rs826472 | T | C | 0.155 | 0.155 | 0.928 | 0.966 |
| 10 | 132,698,419 | rs964681 | C | T | 0.339 | 0.334 | 0.223 | 0.672 |
| 11 | 11,096,221 | rs901398 | C | T | 0.302 | 0.293 | 0.316 | 0.447 |
| 11 | 134,667,546 | rs2076848 | T | A | 0.349 | 0.363 | 0.035 | 0.263 |
| 12 | 23,769,449 | rs10771010 | T | C | 0.457 | 0.457 | 0.097 | 0.998 |
| 12 | 26,171,576 | rs1994997 | A | G | 0.349 | 0.359 | 0.217 | 0.440 |
| 13 | 20,901,724 | rs1335873 | T | A | 0.314 | 0.329 | 0.055 | 0.216 |
| 13 | 106,938,411 | rs354439 | A | T | 0.369 | 0.359 | 0.345 | 0.399 |
| 13 | 108,332,074 | rs9583190 | T | C | 0.427 | 0.418 | 0.961 | 0.465 |
| 14 | 25,850,832 | rs1454361 | A | T | 0.478 | 0.478 | 0.671 | 0.979 |
| 15 | 53,616,909 | rs8037429 | T | C | 0.390 | 0.385 | 0.345 | 0.685 |
| 15 | 91,307,410 | rs2518968 | G | C | 0.353 | 0.372 | 0.207 | 0.142 |
| 16 | 5,606,197 | rs729172 | T | G | 0.132 | 0.124 | 0.828 | 0.363 |
| 16 | 80,106,361 | rs1382387 | C | A | 0.305 | 0.308 | 0.151 | 0.777 |
| 17 | 5,706,623 | rs740910 | G | A | 0.067 | 0.058 | 0.164 | 0.167 |
| 17 | 11,694,706 | rs2010253 | C | T | 0.414 | 0.428 | 0.667 | 0.261 |
| 17 | 49,716,223 | rs1468118 | C | T | 0.341 | 0.357 | 0.246 | 0.204 |
| 18 | 1,127,986 | rs1493232 | A | C | 0.368 | 0.359 | 0.160 | 0.491 |
| 18 | 44,580,082 | rs2247221 | T | C | 0.473 | 0.473 | 0.479 | 0.997 |
| 18 | 75,432,386 | rs1024116 | T | C | 0.086 | 0.077 | 0.618 | 0.181 |
| 20 | 39,487,110 | rs1005533 | A | G | 0.336 | 0.326 | 0.830 | 0.449 |
| 21 | 29,679,687 | rs2831700 | A | G | 0.449 | 0.436 | 0.125 | 0.328 |
| 21 | 42,415,929 | rs914165 | A | G | 0.327 | 0.315 | 0.072 | 0.360 |
| 21 | 47,546,244 | rs13050660 | C | T | 0.354 | 0.369 | 0.155 | 0.241 |
| 22 | 31,160,539 | rs136337 | G | T | 0.346 | 0.336 | 0.136 | 0.417 |
| 22 | 47,836,412 | rs2040411 | A | G | 0.259 | 0.279 | 0.219 | 0.090 |
| 22 | 48,362,290 | rs1028528 | G | A | 0.332 | 0.335 | 0.800 | 0.864 |
